# Hybrid Whale Optimization with a Firefly Algorithm for Function Optimization and Mobile Robot Path Planning

**DOI:** 10.3390/biomimetics9010039

**Published:** 2024-01-08

**Authors:** Tao Tian, Zhiwei Liang, Yuanfei Wei, Qifang Luo, Yongquan Zhou

**Affiliations:** 1 College of Economics, Guangxi Minzu University, Nanning 530006, China; tiantao@gxmzu.edu.cn; 2 College of Electronic Information, Guangxi Minzu University, Nanning 530006, China; 3 College of Artificial Intelligence, Guangxi Minzu University, Nanning 530006, China; 20060043@gxun.edu.cn; 4 School of Information Engineering, Chang’an University, Xi’an 710064, China; 5 Faculty of Information Science and Technology, Universiti Kebangsaan Malaysia (UKM), Bangi 43600, Selangor, Malaysia; weiyuanfei@gxxshxy.edu.cn; 6 Guangxi Key Laboratories of Hybrid Computation and IC Design Analysis, Nanning 530006, China

**Keywords:** whale optimization algorithm, firefly algorithm, opposite-based learning, mobile robot path planning, multi-population, hybrid metaheuristic algorithm

## Abstract

With the wide application of mobile robots, mobile robot path planning (MRPP) has attracted the attention of scholars, and many metaheuristic algorithms have been used to solve MRPP. Swarm-based algorithms are suitable for solving MRPP due to their population-based computational approach. Hence, this paper utilizes the Whale Optimization Algorithm (WOA) to address the problem, aiming to improve the solution accuracy. Whale optimization algorithm (WOA) is an algorithm that imitates whale foraging behavior, and the firefly algorithm (FA) is an algorithm that imitates firefly behavior. This paper proposes a hybrid firefly-whale optimization algorithm (FWOA) based on multi-population and opposite-based learning using the above algorithms. This algorithm can quickly find the optimal path in the complex mobile robot working environment and can balance exploitation and exploration. In order to verify the FWOA’s performance, 23 benchmark functions have been used to test the FWOA, and they are used to optimize the MRPP. The FWOA is compared with ten other classical metaheuristic algorithms. The results clearly highlight the remarkable performance of the Whale Optimization Algorithm (WOA) in terms of convergence speed and exploration capability, surpassing other algorithms. Consequently, when compared to the most advanced metaheuristic algorithm, FWOA proves to be a strong competitor.

## 1. Introduction

Mobile robots are widely used in aerospace, entertainment, agriculture, the military, mining, and rescue operations [1]. They have attracted the attention of many scholars. Meltem Eyuboglu [2] proposed a novel collaborative path planning algorithm for a 3-wheel omnidirectional Autonomous Mobile Robot, Arash Marashian [3] proposed a method for solving mobile robots’ path-planning and path-tracking in static and dynamic environments. Nina Majer [4] proposed a Game-Theoretic Trajectory Planning of Mobile Robots in Unstructured Intersection Scenarios, Guangxin Li [5] solved the path planning of a mobile robot by mixing the algorithms of ACO and ABC. Zhiheng Yu [6] proposed a path planning algorithm for mobile robots based on the water flow potential field method and the beetle antennae search algorithm. De Zhang [7] proposed Multi-objective path planning for mobile robots in nuclear accident environments based on improved ant colony optimization with modified A*, Patrick F. and Charles P. [8] proposed the kinematic modeling of wheeled mobile robots, and Junlin Ou proposed a hybrid path planning based on adaptive visibility graph initialization and edge computing for mobile robots [9]. In many fields of its application, path planning is the most important part. Path planning aims to find a collision-free, optimally safe path from the starting point to the target point in the environment with obstacles according to certain performance indicators, such as planning time, path smoothness, and walking convenience.

Many methods have been applied to mobile robot path planning (MRPP), such as those of Guodong Zhu and Peng Wei, who use dynamic geofencing to solve the path planning problem [10]. Elie Hermand [11] proposes a constrained control scheme to steer an UAV to the desired position while ensuring constraint satisfaction at all times. Joseph Kim and Ella Atkins [12] use airspace Geofencing to solve path planning problems. With the development of research, the swarm-based algorithm has been applied to the MRPP. Unlike traditional algorithms, swarm-based algorithms can perform many intelligent tasks accurately and robustly, which is due to the inspiration of biological intelligence. Therefore, the swarm-based algorithm improves the accuracy of the solution, and lots of scholars use the swarm-based algorithm to solve the MRPP. V. Sathiya [13] proposed a FIMOPSO to solve mobile robot path planning. A. Lazarowska [14] uses the Discrete Artificial Potential Field algorithm (DAPF) to solve the MRPP. Zhang Chungang [15] solved the mobile robot rolling path planning problem. Guangsheng Li [16] uses self-adaptive learning particle swarm optimization to solve the MRPP. These optimization methods show that MRPP has attracted the attention of many scholars (See Table 1).

A swarm-based algorithm is a kind of metaheuristic algorithm. Other metaheuristic optimization algorithms include biological evolution-based, Swarm-based, physical- and chemistry-based, and human-based algorithms. Swarm-based algorithm is a kind of classical algorithm, such as the Hunting search algorithm (HSA) [17], the Grasshopper optimisation Algorithm (GOA) [18], Cat Swarm Optimization (CSA) [19], particle swarm optimization (PSO) [20], Firefly algorithm (FA) [21], Salp Swarm Algorithm (SSA) [22], Whale optimization algorithm (WOA) [23], and gray wolf optimization algorithm (GWO) [24]. Because of its simple concept and remarkable performance, this kind of algorithm is widely studied and applied. Whale optimization algorithm (WOA) [23] is a famous swarm-based algorithm proposed by Mirjalili in 2016. The algorithm solves the problem by simulating the hunting behavior of whales. The hunting process is the optimization process. Because of its remarkable performance in solving problems, the algorithm has been widely studied in the academic community.

The main contributions of this paper are as follows: In order to improve the accuracy of MRPP and WOA’s performance and broaden the application of WOA, a hybrid whale-firefly optimization algorithm based on multi-population and Opposition-Based Learning is proposed in this paper. Firstly, to improve the exploration ability and balance exploitation and exploration, the multiple population mechanism is introduced for the division of labor and cooperation. Secondly, aim to solve the problem of poor accuracy of the algorithm by introducing the Opposition-Based Learning (OBL) and improving the optimization ability of the algorithm through symmetric mapping. The performance of the algorithm is improved by the above two methods. On this basis, in order to better conform to the biological mechanism, the Perception of the food population of whales is introduced to expand the search space and further improve the exploration ability.

The rest of this paper is set as follows: Section 2 introduces the classical whale optimization algorithm. Section 3 introduces the FWOA. Section 4 introduces the verification of FWOA, and Section 5 introduces the MRPP model. Section 6 describes the simulation results and analysis. Section 7 contains conclusions and future work.

## 2. Whale Optimization Algorithm

Whale optimization algorithm (WOA) is based on the hunting behavior of humpback whales. It mainly includes three phases: Encircling prey, Bubble-net attacking method (exploitation phase), and Search for prey.

### 2.1. Encircling Prey

Humpback whales can locate their prey and encircle them. The WOA defines the current best candidate solution as the best solution [23]. The other search agents will update the position toward the leader whales (the best solution defined); the equations of this behavior are expressed as follows:

(1) $$\vec{D}=|\vec{C}•{\vec{X}}^{*}(t)-\vec{X}(t)|$$

(2) $$\vec{X}(t+1)={\vec{X}}^{*}(t)-\vec{A}•\vec{D}$$

where *t* is the current iteration, $\vec{A}$ and $\vec{C}$ are coefficient vectors, ${\vec{X}}^{*}$ indicates the position vector of the best solution obtained so far, $\vec{X}$ is the position vector, | | is the absolute value, and • is an element-by-element multiplication. Notes that ${\vec{X}}^{*}$ should be updated in each iteration if there is a better solution.

The vectors $\vec{A}$ and $\vec{C}$ are calculated as follows:

(3) $$\vec{A}=2\vec{a}•\vec{r}-\vec{a}$$

(4) $$\vec{C}=2•\vec{r}$$

The $\vec{a}$ is linearly decreased from 2 to 0 over the course of iterations and $\vec{r}$ is a random vector in [0,1].

### 2.2. Bubble-Net Attacking Method (Exploitation Phase)

Bubble-net attacking method includes the shrinking encircling mechanism and the spiral updating position method. The search agents will choose a method to update their position.

Shrinking encircling mechanism: By decreasing the value of $\vec{a}$ in Equation (3), whales can shrink and encircle prey [23]. Spiral updating position: This mechanism firstly calculates the distance between the whale position and the prey position, then, by establishing an equation between the search agents and the prey, the update of position is achieved. To mimic this method, the equations are as follows:

(5) $$\vec{D^{\prime }}=|\vec{C}•{\vec{X}}^{*}(t)-\vec{X}(t)|$$

(6) $$\vec{X}(t+1)=\vec{D^{\prime }}•e^{bl}•\cos(2\pi l)+{\vec{X}}^{*}(t)$$

The $\vec{D^{\prime }}$ is the distance of the *i*th whale to the prey, *b* is a constant for defining the shape of the logarithmic spiral, *l* is a random number in [−1, 1], and • is an element-by-element multiplication.

A parameter *p* is introduced to control the switch between the shrink encircling mechanism and the spiral updating position method. The equation is as follows:

(7) $$\vec{X}(t+1)=\left\{\begin{array}{l} {\vec{X}}^{*}(t)-\vec{A}•\vec{D}\\ \vec{D^{\prime }}•e^{bl}•\cos(2\pi l)+{\vec{X}}^{*}(t) \end{array}\right.\;\;\;\;\begin{array}{l} if\begin{array}{cc} p<0.5 & \end{array}\\ if\begin{array}{cc} p\geq 0.5 & \end{array} \end{array}$$

The *p* is a random number in [0,1].

### 2.3. Search for Prey

For the exploration phase, agents update the position by randomly selecting whales. The random value of *A* that is greater than 1 or less than −1 can let them move far away from the prey. This mechanism and |*A*| < 1 together let the algorithm perform a global search. The equations are expressed as follows:

(8) $$\vec{D}=|\vec{C}•{\vec{X}}_{\mathrm{ran}d}-\vec{X}|$$

(9) $$\vec{X}(t+1)={\vec{X}}_{rand}(t)-\vec{A}•\vec{D}$$

where ${\vec{X}}_{rand}(t)$ indicated a random position vector chosen from the current population.

The WOA algorithm starts with a random population and then updates the solution at each iteration. While the condition is satisfied, the algorithm concludes that the solution is the best solution.

## 3. The Proposed FWOA

Based on classical WOA, this section proposes a hybrid whale-firefly optimization algorithm based on multi-populations and Opposition-Based Learning. The FWOA has three improvements: multi-populations, hybrids with the firefly algorithm (FA), and the perception of food.

### 3.1. The Multi-Populations

Like primitive humans, all social creatures will divide and cooperate according to the task type. The division and cooperation of ants ensure the stability of their society, and the division and cooperation of wolves ensure the efficiency of hunting prey. Research shows that the whale will also carry out division and cooperation, dividing the total population into several subpopulations. Each subpopulation has its own task, and the entire whale population will predate in this way.

In order to make the algorithm more consistent with the natural mechanism and improve its performance while balancing its exploitation and exploration, this paper divides the initial whale population into two subpopulations: (1) the Search Population (SP) and (2) the Hunt Population (HP). The number of whales in each population accounts for half of the total population. Assign different tasks to different populations to achieve the goal of division of labor and cooperation.

The main task of the search population (SP) is to search (exploration). Through its fast exploration of the search space, it can find the region that is most likely to have the optimal solution. After each search, it will continue to look for other possible locations for the best solution. Through this mechanism, the exploration ability of the algorithm is greatly improved, which enables the algorithm to quickly find the location of the optimal solution.

The main task of the hunt population (HP) is to hunt (exploitation). After the search population has locked down the optimal value area, the hunt population will be exploited in this area to find the optimal value. This mechanism ensures the exploitation ability of the classic WOA. Furthermore, different tasks make the two populations focus on different aspects at the same time. The hunt population focuses on exploitation, and the search population focuses on exploration, realizing the balance between exploitation and exploration. The tasks of the hunt population and search population in different phases are described as follows:

#### 3.1.1. Search Prey

In the search for prey phase, in order to reflect the independence between populations, two populations randomly select a leader whale from their own populations and update the position according to the position of their own leader whale. The position update method for the search population is as follows:

(10) $${\vec{D}}_{s}=|\vec{C}•{\vec{X}}_{r,s}-{\vec{X}}_{s}|$$

(11) $${\vec{X}}_{s}(t+1)={\vec{X}}_{r,s}(t)-\vec{A}•{\vec{D}}_{s}$$

where ${\vec{D}}_{s}$ is the distance between the current whale and the leader whale randomly selected in the search population, ${\vec{X}}_{r,s}$ is the leader whale selected in the search population, and ${\vec{X}}_{s}$ is the position of the whale in the search population.

The position update method for the hunt population is as follows:

(12) $${\vec{D}}_{h}=|\vec{C}•{\vec{X}}_{r,h}-{\vec{X}}_{h}|$$

(13) $${\vec{X}}_{s}(t+1)={\vec{X}}_{r,h}(t)-\vec{A}•{\vec{D}}_{h}$$

where ${\vec{D}}_{h}$ is the distance between the current whale and the leader whale randomly selected in the hunt population, ${\vec{X}}_{r,h}$ is the leader whale selected in the hunt population, and ${\vec{X}}_{r,h}$ is the position of the whale in the hunt population.

#### 3.1.2. Encircling Prey

In the encircling prey phase, in order to reflect the cooperation of the population and to improve the efficiency of the algorithm, the search population and the hunt population in this phase are merged to form a combined population (CP). The search method for combined population (CP) is according to the phase of Encircling prey in classic WOA. In this phase, the combination of populations is realized, thus improving the computational efficiency of the algorithm. The position update method for the population is as follows:

(14) $${\vec{D}}_{c}=|\vec{C}•{\vec{X}}^{*}(t)-{\vec{X}}_{c}(t)|$$

(15) $${\vec{X}}_{c}(t+1)={\vec{X}}^{*}(t)-\vec{A}•{\vec{D}}_{c}$$

where ${\vec{D}}_{c}$ is the distance between the current whale and the best whale in the combined population, ${\vec{X}}^{*}$ is the position of the leader whale, and ${\vec{X}}_{c}$ is the position of the current whale.

#### 3.1.3. Bubble-Net Attacking Method (Exploitation Phase)

In contrast to the above phases, in the bubble-net attacking method, the two populations were assigned different tasks. To emphasize the exploration behavior, the search population first randomly selects a leader whale from the search population, and other whales in the population update the position of the whale to perform a search behavior to improve the exploration ability of the algorithm. The method is expressed as Equation (10).

The hunt population uses the position update method of classical WOA, and it is as follows:

(16) $${\vec{D}}_{h}=|{\vec{X}}^{*}(t)-{\vec{X}}_{h}(t)|$$

(17) $${\vec{X}}_{h}(t+1)={\vec{D}}_{h}•e^{bl}•\cos(2\pi l)+{\vec{X}}^{*}(t)$$

where ${\vec{D}}_{h}$ is the distance between the current whale and the best whale, ${\vec{X}}^{*}(t)$ is the best whale position, and ${\vec{X}}_{h}(t+1)$ is the position of the hunt population.

### 3.2. Bubble-Net Attacking Method

#### 3.2.1. The Perception of Food

Nature is full of magic. Spider sensing can help spiders avoid danger. Like spiders, studies have found that whales also have a perception, but it is the perception of food, which may be based on smell or temperature. This perception allows whales to quickly explore areas where food may exist when hunting, improve hunting efficiency, and provide more food for the whales. Compared with the excellent exploitation ability of classical WOA, its exploration ability is slightly inferior. Due to its unique exploration mechanism, WOA does not have a good search direction during exploration but randomly selects the direction. Although this exploration mechanism provides good randomness for the algorithm, it shows a relatively inferior ability in terms of efficiency. To improve this weakness, this section applies the whale’s perception ability to classical WOA to improve the exploration ability and efficiency of classic WOA.

The focus of food perception is to guide whales in the direction of predation, so after each iteration of the algorithm, the entire population will conduct a food perception. Through this perception, the optimal population search direction will be found. At the same time, in order to ensure the randomness of the algorithm and avoid getting stuck at local optimal, the perception direction of the optimal population will be compared with the current optimal searcher after each perception, and the best one of the two will be found, and this direction will become the position update direction of the entire population, so as to improve the exploration ability and enable the algorithm to quickly find the optimal value.

#### 3.2.2. Firefly Algorithm (FA)

The Firefly algorithm (FA) [21] was proposed by Xin She Yang in 2008. It is an idealized behavior based on the flicker characteristics of fireflies [25]. There are several important parameters in the firefly algorithm: (1) Light intensity and attraction β ; (2) Firefly in horizontal position *x*; (3) Firefly in vertical position $y_{i}$ ; (4) Distance between firefly *i* and *j* $r_{ij}$ ; (5) Intensity of light source $I_{s}$ .

The brightness of the firefly at a certain position or position x (represented by I ) can be calculated as follows:

(18) I(x)αf(x)

The attraction must be adjusted as a function of absorption. Thus, the change in light intensity I(r) follows the inverse square law:

(19) $$I(r)=\frac{I_{s}}{r^{2}}$$

Meanwhile, consider the static light absorption coefficient γ ; the intensity I of light varies with position or distance r , thus:

(20) $$I=I_{0}e^{-\gamma r}$$

where $I_{0}$ indicates the actual intensity of light.

The attraction of fireflies β can be approximated as:

(21) $$\beta =\frac{\beta_{0}}{\left(1+\gamma r^{2}\right)}$$

where $\beta_{0}$ is the attractiveness level when r=0 .

Then, the distance between two fireflies can be calculated. Let firefly *i* and firefly *j* be $x_{i}$ and $x_{j}$ on the horizontal axis, and on the vertical $y_{i}$ $y_{j}$ axis, the distance between them can be calculated as:

(22) $$r_{i,j}=\sqrt{{\left(x_{i}-x_{j}\right)}^{2}+{\left(y_{i}-y_{j}\right)}^{2}}$$

As mentioned above, the navigation of firefly *i* is attracted by another highly attractive firefly *j*, and the movement of firefly *i* towards firefly *j* is expressed mathematically as follows:

(23) $$x_{i}=x_{i}+\beta_{0}e^{-\gamma r_{i,j}^{2}}(x_{j}-x_{i})+\alpha (rand-\frac{1}{2})$$

where *rand* is a random number in the interval [0,1], α . The coefficient of the random displacement vector, γ the light absorption coefficient of the environment, $r_{i,j}$ and the Euclidean distance between two fireflies.

For the maximization problem, the brightness can be simply proportional to the objective function. Other forms of brightness can be defined in a way similar to the fitness function in a genetic algorithm or bacterial foraging algorithm (BFA) (Algorithm 1).
**Algorithm 1** Pseudocode of the Firefly AlgorithmDefine target function which is presented as: 
$f(x):x=(x_{1},x_{2},\ldots ,x_{d})$
Generate or develop preliminary or pilot population of fireflies: 
$x_{i}(i=1,2,\ldots ,n)$
Define expression for intensity of light (1) so that it is linked with 
I=f(x)
Define the light adsorption, represented by *y***While** (*t* < maximum generation of light) **For** 
i=1:n
 (for all fireflies in the sample space)  **For** 
j=1:n
 (for all fireflies in the sample space)   **If** (
$I_{j}>I_{I}$
)    Firefly *i* move towards firefly *j*
   **End if**
   Express attractiveness of firefly based on the separation point (*r*) distance 
$\exp(-yr^{2})$
   Estimate original value and present the final value in terms of light intensity
  **End for**

 **End for**
 Find the best possible firefly
**End while**


#### 3.2.3. The Hybrid of WOA and FA

Similar to fireflies’ perception of light, food perception is a whale’s ability, so in the food perception phase, let the search agent perceive according to the FA method to find the optimal food direction. At the beginning of perception, everyone in the population randomly generates a positional perception of food. *Position* food is not perceived according to the current position of the individual, which is set to ensure the randomness of the individual population. In order to improve the performance of the algorithm, a probability selection is made during food perception to make the algorithm targeted. Therefore, a random number *q* is generated after the random food location is generated. If the value of the random number *q* is less than 0.5, the food perception is updated as follows:

(24) $$x_{f}=x_{f}+\beta_{0}e^{-\gamma r_{i,j}^{2}}(x_{f,j}-x_{f,i})+\alpha (q-\frac{1}{2})\;\;\;if\begin{array}{cc} q<0.5 & \end{array}$$

where $r_{i,j}$ is the Euclidean distance between food perception location *i* and food perception location *j*, and other parameters are as shown above.

If the value of *q* is greater than 0.5, a random position perception is performed to regenerate a new food position, so as to greatly improve the randomness of the algorithm while ensuring performance improvement and keeping the algorithm in a steadily improving state.

### 3.3. Opposition-Based Learning

Opposition-Based Learning is a strategy proposed by Hamid R. tizhoosh in 2005 [26]. The main idea of this strategy is: when people are solving the solution *x* of a given problem, they usually need to estimate a solution $\tilde{x}$

In many cases, learning starts at random points (initialization of the population). In algorithms, it starts with a random population and moves the solution towards the optimal solution. Based on this thinking, it is beneficial to improve the efficiency of the algorithm if the opposite number $\tilde{x}$ is calculated when searching for *x*

Suppose x∈R,x∈[a,b] . The opposite number $\tilde{x}$ of *x* is calculated as follows:

(25) $$\tilde{x}=a+b-x$$

The formula is extended to the multi-dimensional case. $x_{i}\in R,x_{i}\in [a_{i},b_{i}]$ . Defined, the equation is as follows:

(26) $${\tilde{x}}_{i}=a_{i}+b_{i}-x_{i}\;\;\;i=1,2,\ldots ,n$$

When it comes to FWOA, $a_{i}$ which $b_{i}$ is the lower bound and the upper bound of the problem, and $x_{i}$ the search agents, the equation is as follows::

(27) $${\tilde{x}}_{p}=lb+ub_{i}-x_{i}\;\;\;i=1,2,\ldots ,n$$

where ${\tilde{x}}_{p}$ is the opposite population of search agents.

**Figure 1:**
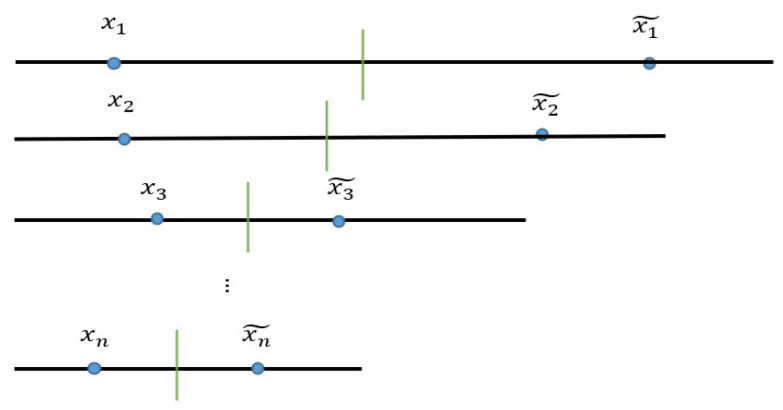
Opposition-based learning.

Figure 1 shows the computer system for antisymmetric learning. Based on this mechanism, the detection ability of the algorithm can be improved, and the traversal of the search space by the algorithm can be increased.

The Pseudocode of the FWOA is as follow (Algorithm 2):
**Algorithm 2** Pseudocode of the FWOAInitialize the whale populations: The search population 
$x_{s}$
 and The hunt population: 
$x_{h}$
Calculate the fitness of each search agent
$X^{*}$
 = the best search agent**while** (***t*** < maximum number of iterations) for each search agent  Update 
a,A,C,l
,
p
   **if1** (
p<0.5
)    **if2** (
|A|<1
)     The combined population 
$x_{c}$
 updates the position of the current search agent by the Equation (15).    **else if2** (
|A|≥1
)     Search population 
$x_{s}$
 selects a random search agent 
$x_{r,s}$
 by Equation (10)     Search population 
$x_{s}$
 updates the position of the current search agent by the Equation (11).     Hunt population 
$x_{h}$
 selects a random search agent 
$x_{r,h}$
 by Equation (12)     Hunt population 
$x_{h}$
 updates the position of the current search agent by the Equation (13)   **end if2**
  **else if1** (
p≥0.5
)   Search population 
$x_{s}$
 selects a random search agent 
$x_{r,s}$
 by Equation (10)   Search population 
$x_{s}$
 updates the position of the current search agent by the Equation (11)   Hunt population 
$x_{h}$
 updates the position of the current search agent by the Equation (17)
  **end if1**

 **end for**
  Initialize the perception of food population 
$x_{f}$
  Update 
q
   **If3** (
p<0.5
)    The combined population 
$x_{c}$
 updates the perception of food position by Equation (24)    **Else if3** (
p≥0.5
)    Initialize a new position of food
   **end if3**
 Find the opposite population 
${\tilde{x}}_{p}$
 by Equation (27) Check if any search agent goes beyond the search space and amend it Calculate the fitness of each search agent Update 
$X^{*}$
 if there is a better solution

 t=t+1


**End while**
Return 
$X^{*}$


## 4. Verification of FWOA

In this section, the FWOA algorithm has been tested on 23 benchmark functions. The 23 benchmark functions are classical functions used by many researchers [27,28,29,30,31]. Although these functions are simple, we chose them to compare our algorithm with the current metaheuristic method to verify the performance of FWOA. Table 2, Table 3 and Table 4 list these benchmark functions. Generally speaking, the reference functions used can be divided into three groups: Uni-modal functions, Multi-modal functions, and Fixed-dimension multi-modal functions. Table 2, Table 3 and Table 4 show these groups of functions, respectively. Different types of functions place different emphasis on performance. Dimension in the table represents the dimension of the function, Range is the boundary of the function search space and $f_{\min}$ is the best value.

### 4.1. Experiment Setting

**Figure 2:**
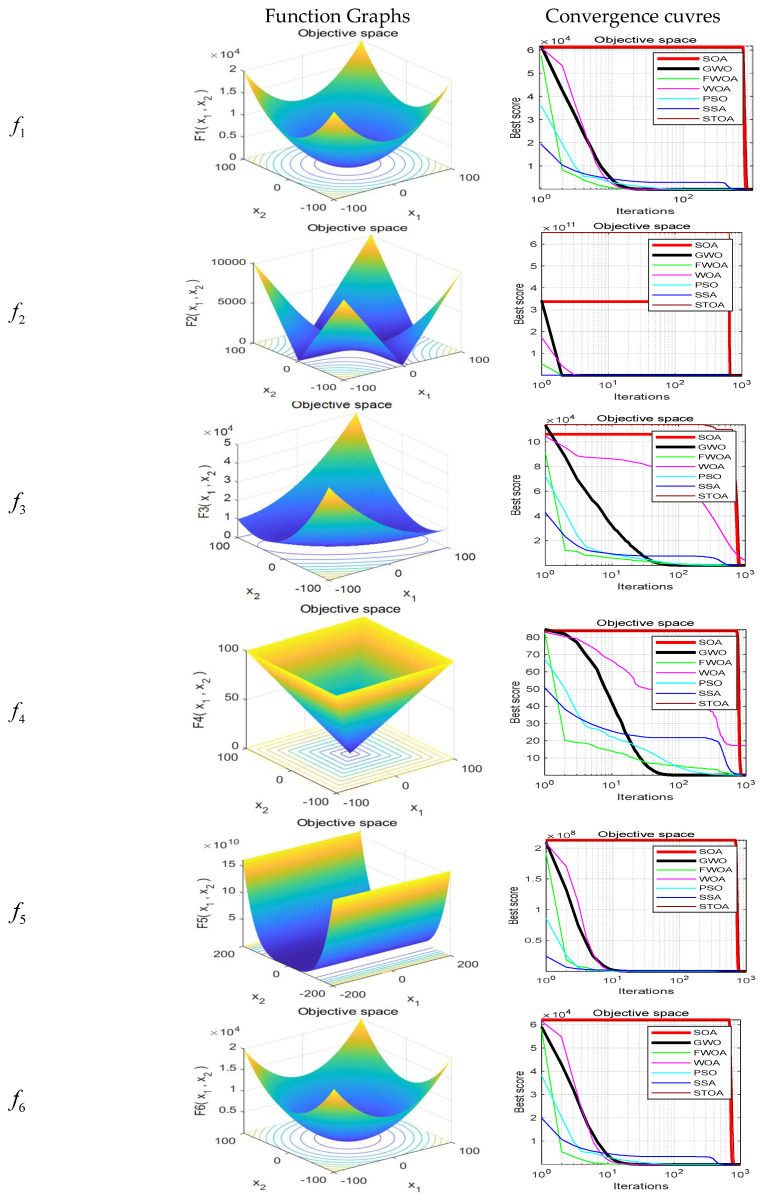

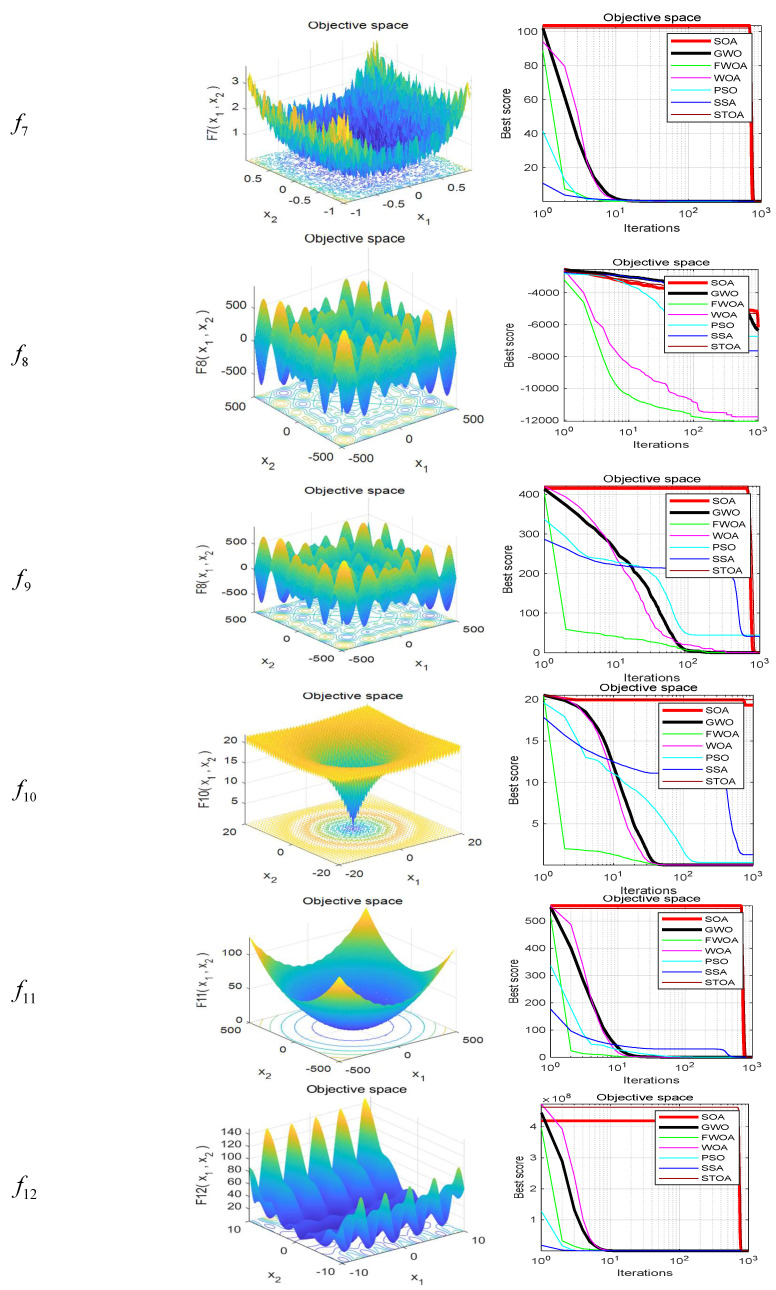

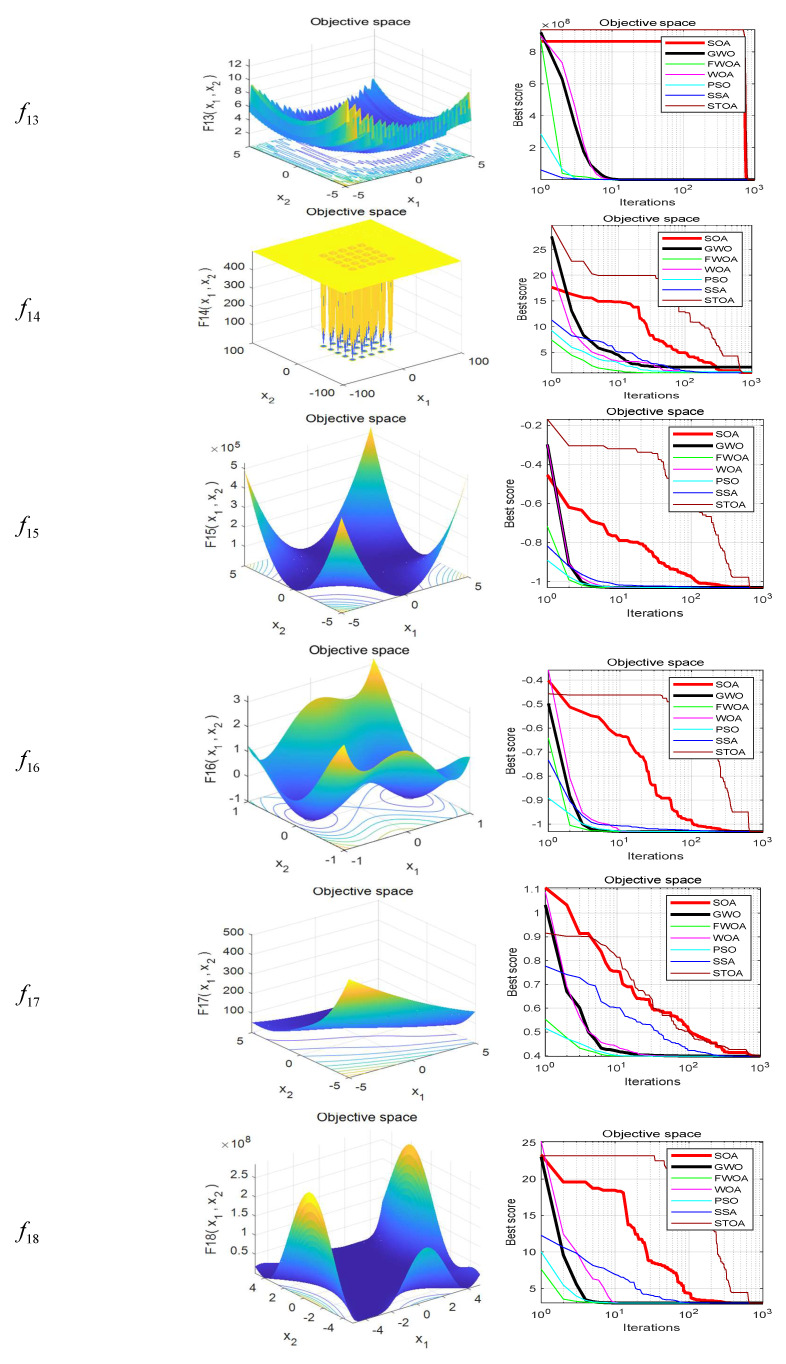

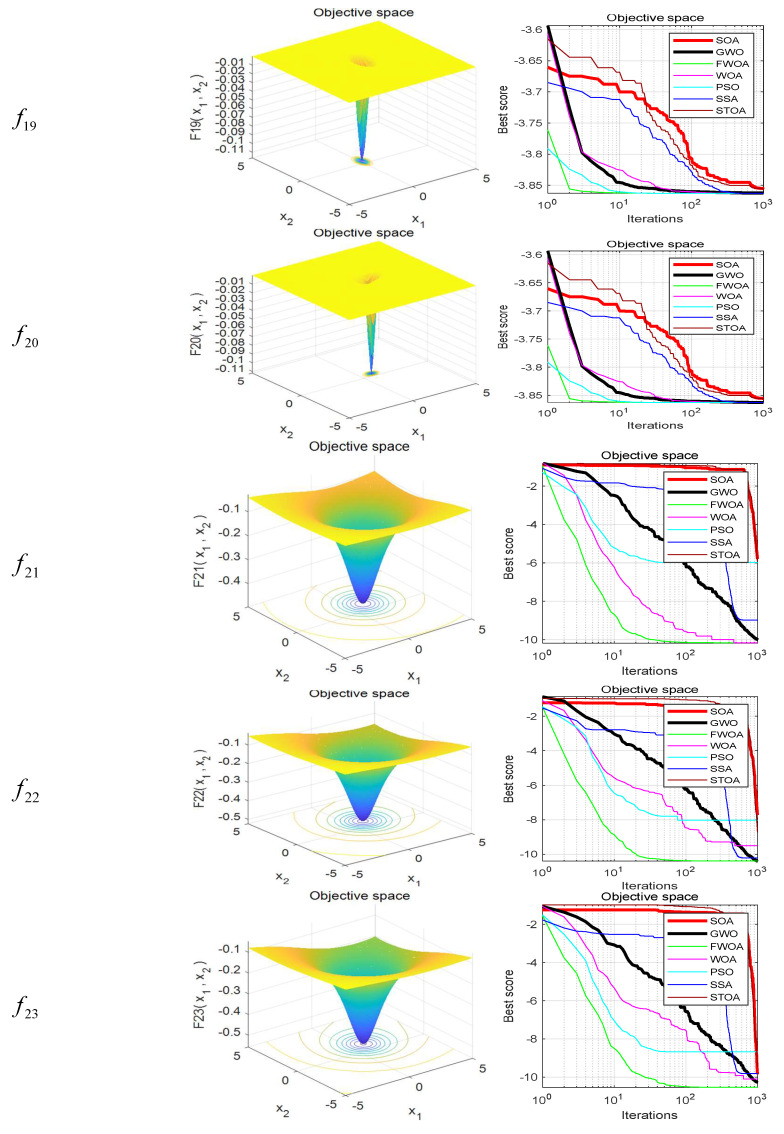
Test functions convergence curves.

The maximum number of iterations of the algorithm is 1000, and the number of search agents is 100. Each algorithm runs independently on each benchmark function 30 times. In order to verify the results, the FWOA algorithm is compared with classic PSO [20], SSA [22], WOA [23], GWO [24], STOA [32], and SOA [33]. The statistical results (average, minimum, maximum, and standard deviation) are shown in Table 5, Table 6 and Table 7. The function graphs and algorithm convergence graphs are shown in Figure 2.

### 4.2. Exploitation Analysis

According to the results in Table 5, FWOA can provide very competitive results. This algorithm is superior to other algorithms in $f_{1}-f_{7}$ . It should be noted that unimodal functions focus on benchmark exploitation. Therefore, these results show that FWOA has better performance in finding the optimal value of the function. This is due to the food perception mechanism discussed earlier.

### 4.3. Exploration Analysis

Compared with unimodal functions, multi-modal functions have many local optimal values, and their complexity grows exponentially with the dimension, so the requirements for algorithm performance of multi-modal functions are stricter. Therefore, they are suitable for benchmarking the exploration abilities of algorithms.

According to the results in Table 6, FWOA can also provide very competitive results on Fixed-dimension multi-modal functions. The FWOA is superior to other algorithms in most functions $f_{8}-f_{12},f_{14}-f_{16},f_{20}-f_{23}$ . This phenomenon is reflected in the fact that FWOA can find the best value smaller than the results of all test algorithms, and the maximum value found by FWOA is also the smallest of all algorithms. In addition, compared with GWO and PSO, which have good exploration capabilities, FWOA shows remarkable performance and can often surpass them. These results show that the FWOA algorithm has certain research value.

### 4.4. The Standard Deviation Analysis

The standard deviation is the arithmetic square root of the variance. The standard deviation can reflect the degree of dispersion of a data set. It is most commonly used in probability statistics as a measure of the degree of statistical distribution. A large standard deviation represents a large difference between most values and their average values; a small standard deviation means that these values are close to the average value, so the difference between the data are small. The smaller the standard deviation in algorithm analysis, the better the stability and robustness of the algorithm.

According to the results in Table 5, Table 6 and Table 7, the standard deviation of FWOA is the smallest in most cases, which means that FWOA has strong stability and can provide a relatively stable calculation. This is due to the multi-population mechanism of the algorithm. The division and cooperation of different populations enable the algorithm to achieve the balance between development and detection, so it can also ensure its stability while maintaining good performance.

### 4.5. The Convergence Analysis

This section shows the convergence of the FWOA. According to Digalakis [28], in the initial step of optimization, the movement of search agents should undergo mutation, which helps metaheuristics widely explore the search space. Then, these changes should be reduced to emphasize exploitation at the end of optimization. To observe the convergence behavior of the FWOA algorithm, the convergence graph of the algorithm is shown in Figure 2. In most cases, FWOA converges first, due to the search population discussed before.

To sum up, compared with the well-known metaheuristic algorithm, the experimental results verify the performance of the FWOA algorithm in solving various benchmark functions. In order to further study the performance of the proposed algorithm, a practical problem (two different problem environments) is used in the following section.

The algorithm is compared with different well-known algorithms to verify its effectiveness.

## 5. Using FWOA to Solve the Mobile Robot Path Planning Problem

The mobile robot path planning problem (MRPP) is a famous research problem. There are lots of different methods to solve it, such as Zhang Z [34] who proposed a method based on A-star and Dijkstra Algorithm, Z Cen [35] who proposed a method based on genetic algorithms and the A* algorithm; Y Lü [36] who proposed a method based on a directional relationship with uncertain environmental information; and Y Cheng [37] who proposed a distributed snake algorithm for mobile robot path planning with curvature constraints. Kurihara K [38] proposed a mobile robot path planning method with the existence of moving obstacles; Msg A [39] proposed an intelligent approach for autonomous mobile robot path planning based on an adaptive neuro-fuzzy inference system; and Zhang Z [40] proposed a method based on the dynamic movement primitives library.

The experimental environment of this study is divided into two parts: (1) The irregular obstacle environment with no influence range; (2) The regular obstacle environment with influence range The irregular obstacle environment with no influence range simulates the shapes of different obstacles in the real environment, and the robot searches for the optimal path to avoid collision in this environment. The obstacle of the regular obstacle environment experiment with influence range is circular. This environment simulates the real environment in which objects of different shapes will produce an influence range. When the robot approaches, there may be different degrees of collision, resulting in different motion conditions. (1: Do not affect the robot’s motion. 2: Slightly affect the motion. 3: Collision to immovable). The influence range of obstacles in this environment is subject to the center of the circle. The influence decreases linearly with the distance between the robot position and the center of the circle, so as to simulate the real environment. The method of solving MRPP by FWOA is introduced as follows:

### 5.1. Irregular Obstacles Environment with None Influence Range

Mobile robot path planning is an important task of intelligent robot research. The first step is to model the environment. In an obstacle-free environment, this paper uses the grid method to model. The grid method decouples the workspace into several simple areas to establish an environment model that is convenient for compute path planning; in this way, the physical space is mapped into an abstract space. The free grid point is represented by 0 , and the obstacle point is represented by 1 . Through this mechanism, the modeling of irregular obstacles can be realized, and it is also convenient for computation.

**Figure 3:**
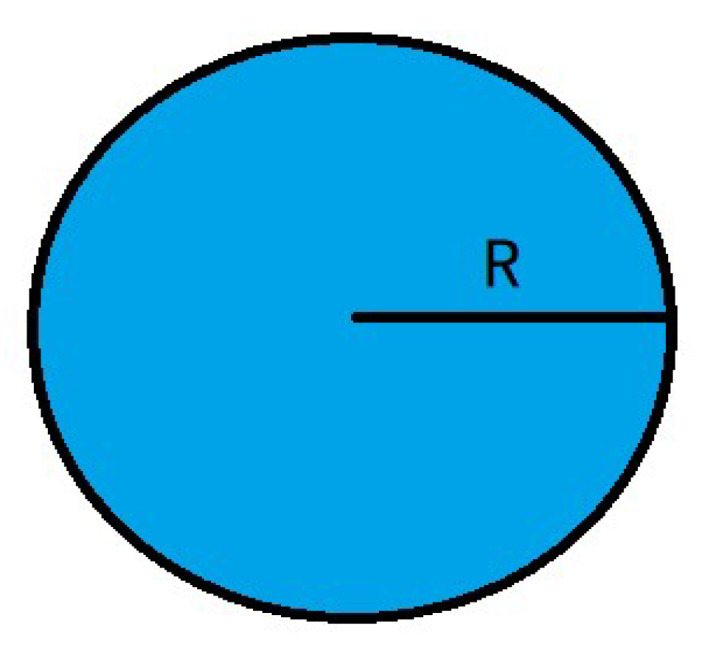
Mobile robot size.

On the two-dimensional map, as shown in Figure 3. in order to solve the path planning problem, we make the following assumptions: (1) The mobile robot only moves in the set search space; (2) There are n different-shape static irregular obstacles in the robot motion space, which are described by the grid method. The obstacles have no influence range, and the path is unavailable when the robot hits the obstacles. (3) Mobile robot is regarded as a particle [41], and its size is ignored. According to the above assumptions, the obstacles are expanded to $R_{s}$ . This $R_{s}$ is obtained by:

(28) $$R_{s}=R+\sigma$$

where σ is the safe distance, which is artificially selected to prevent the mobile robot from contacting obstacles.

**Figure 4:**
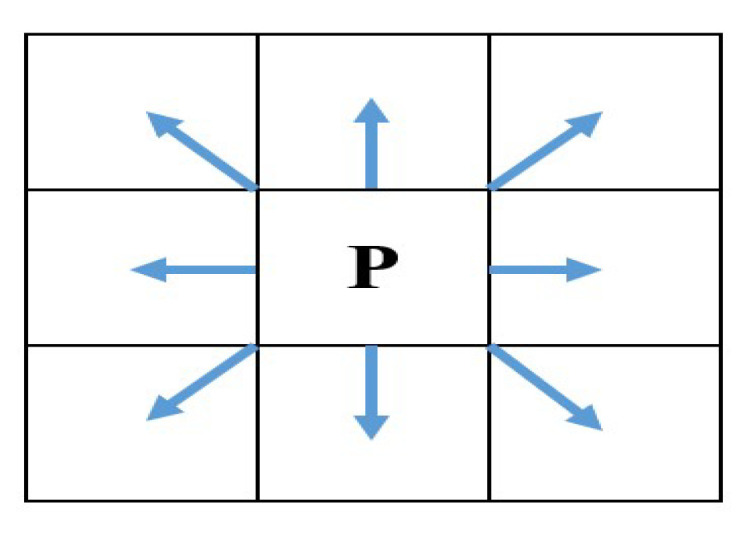
The directions of mobile robot.

The robot moves in eight directions, as shown in Figure 4. Through different moving directions, we can realize the path planning of the mobile robot when moving to any grid point in the search space [41]. The cost function of this model is the motion distance of the robot in two-dimensional space.

### 5.2. Regular Obstacle Environment with Influence Range

Due to the irregular shape of obstacles, their influence ranges are different. When a robot encounters an obstacle while moving in the environment, three situations may occur: (1) stop moving; (2) affect but do not stop moving; and (3) do not affect moving. Based on these situations, this section introduces the obstacle environment with a regular influence range that is more realistic.

The path planning problem in this environment is to find a connection between the starting point and the target point with the least threat, as shown in Figure 3. The point S is the starting point, and the point t is the target point. In order to simplify the problem, the general problem is divided into several sub-problems by using the deconstruction method. Thus, the starting point and the target point are connected by a line, and the connection is divided into 𝑚 segments. The path planning is carried out for each segment, and the path length is the sum of the subpaths.

In [42], an obstacle probability density model based on UAV movement is introduced. The model describes that the influence range of obstacles will not have a boundary when the UAV is moving but will decrease with the increase in distance between the UAV and the obstacle center, but will never be zero. Based on this theory, the probability density model is proposed as follows:

(29) $$C_{\inf luence}=\exp(-\frac{\sum_{i=1}^{n}\left||d_{i}|\right|}{\delta })$$

where δ is a parameter that controls the shape of the density function, $\left||d_{i}|\right|$ indicates the distance from the moving object to the *i*th bstacle.

**Figure 5:**
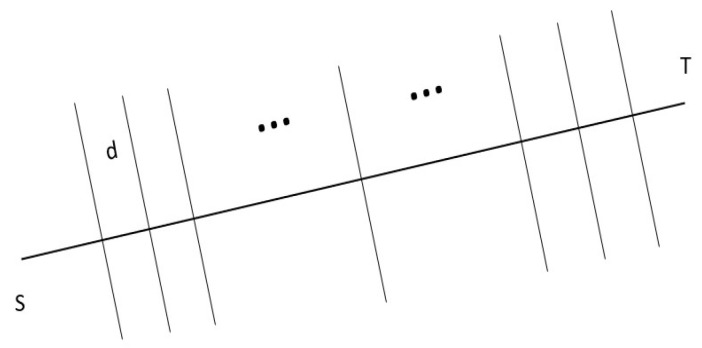
The method of solving.

For the robot, it can be known that the impact of obstacles in the environment on the robot also decreases with the distance from the center of the obstacle, so the model can be used to model the robot path planning problem. However, collision has a great impact on the robot to a certain extent, which is much greater than the impact of obstacles on the UAV. But the probability density value drops too fast to truly simulate the environment. In order to solve this problem, the probability model is improved as follows. Figure 5 shows the improved probability density value, which is smoother than the original probability density curve:

(30) $$C_{\inf lience}=\sqrt{\exp(-\frac{\sum_{i=1}^{n}\left||d_{i}|\right|}{\delta }})$$

The descent speed of the improved probability model becomes slower, which is more in line with the robot situation. Based on this probability density model and in combination with the path planning model in article [3], let the parameter *D* be the length of the motion path, the parameter *S* be the distance of the subproblem segment, and the parameter w be the weight. The following model is proposed to find the shortest distance while considering the influence range:

(31) $$C=(C_{\inf luence}•w+\frac{D}{S}•(1-w))$$

Based on this objective function, the path planning problem can be modeled to solve this problem.

## 6. Simulation Results and Analysis

This section introduces the simulation experiment setting and the analysis of the experiment. The algorithm is tested in ten different mobile robot working environments, and the results show that FWOA is very competitive.

### 6.1. Experimental Setting

In order to evaluate the quality of the algorithm, FWOA is applied to various mobile robot working environments. The experiment was divided into two groups: (1) Irregular obstacles environment with no influence range; and (2) Regular obstacle environment with influence range.

**Figure 6:**
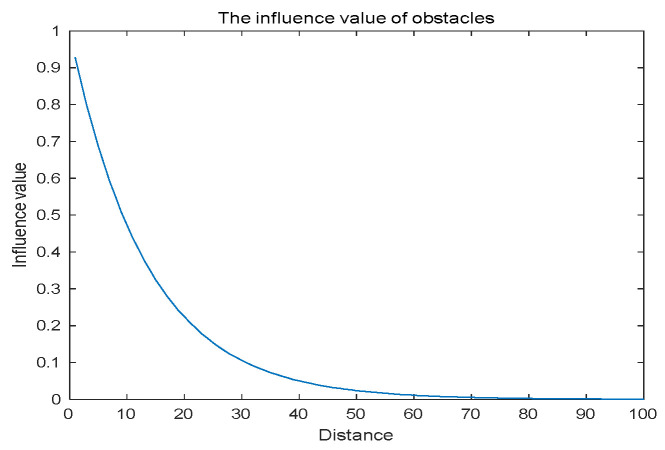
Modified $C_{influence}$ .

**Figure 7:**
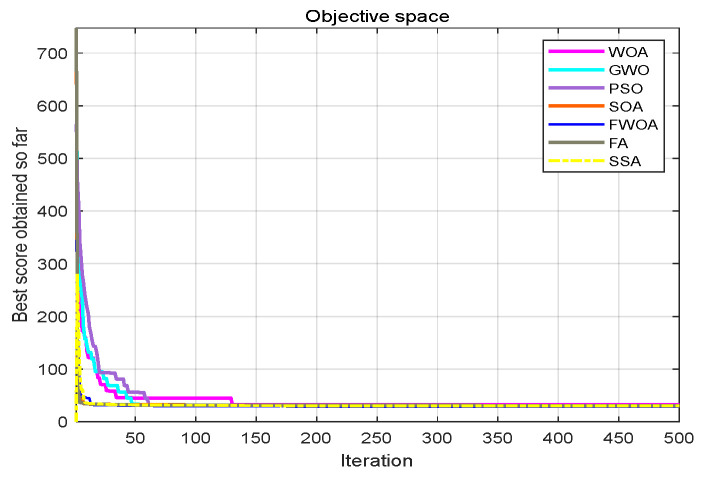
The convergence graph of environment 1.

**Figure 8:**
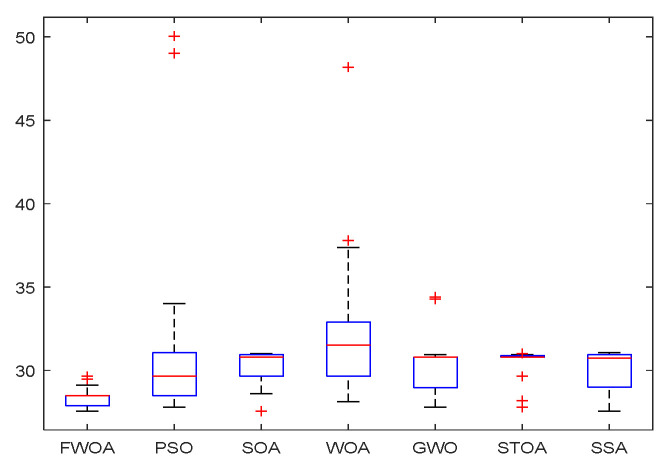
The Boxplot of Environment 1.

**Figure 9:**
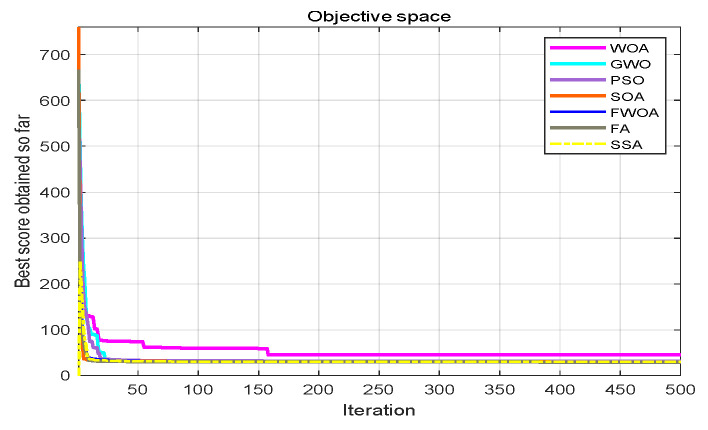
The convergence graph of environment 2.

**Figure 10:**
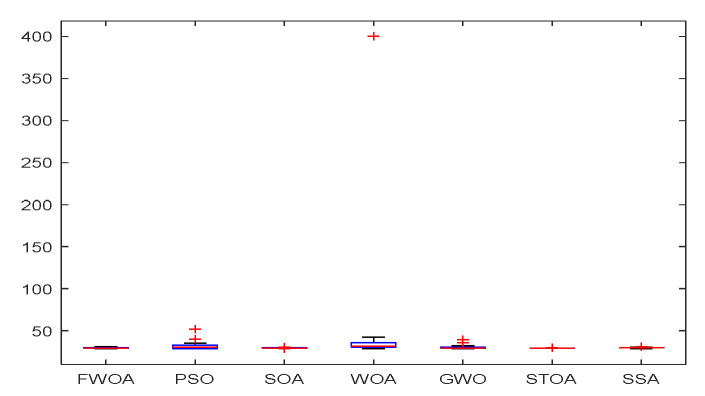
The Boxplot of Environment 2.

**Figure 11:**
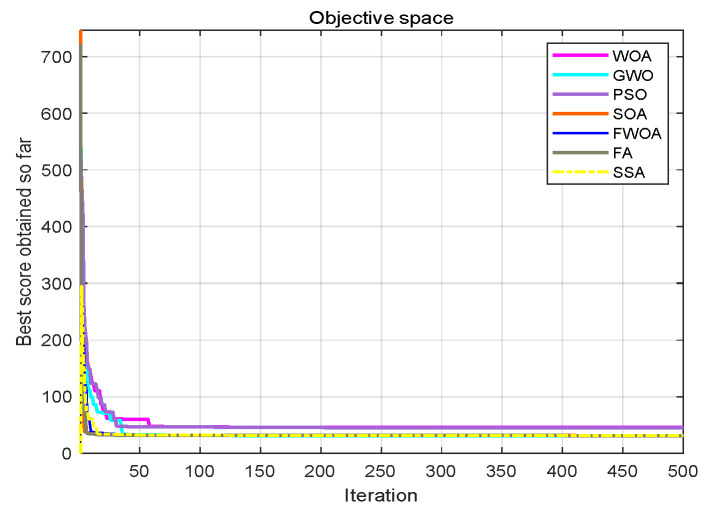
The convergence graph of environment 3.

**Figure 12:**
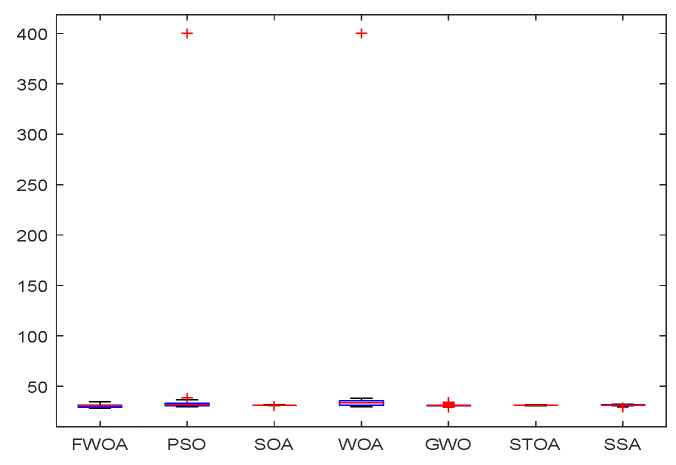
The Boxplot of Environment 3.

**Figure 13:**
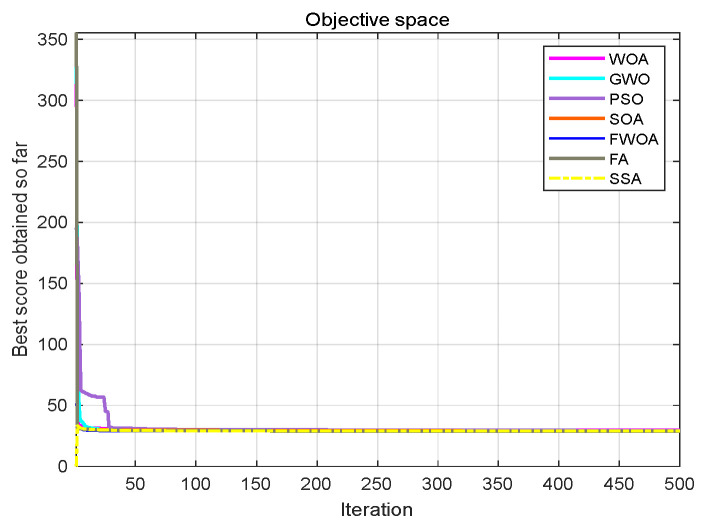
The convergence graph of environment 4.

**Figure 14:**
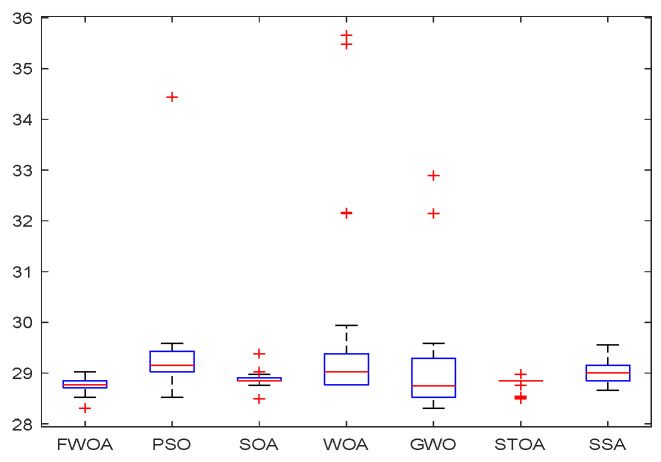
The Boxplot of Environment 4.

**Figure 15:**
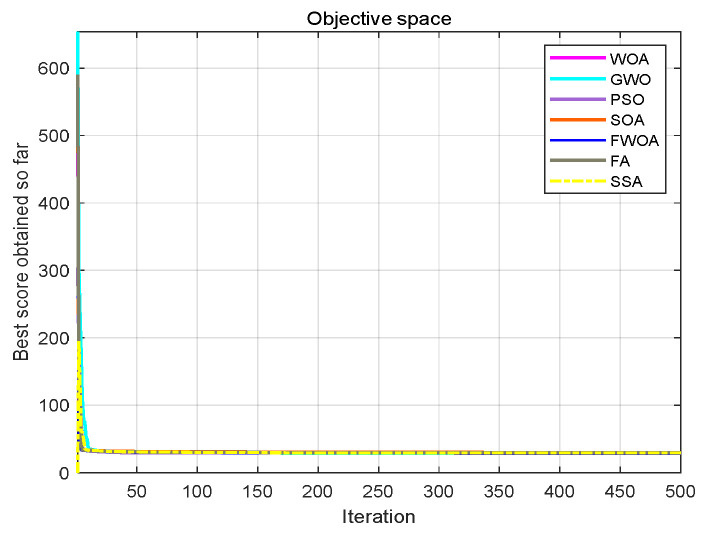
The convergence graph of environment 5.

**Figure 16:**
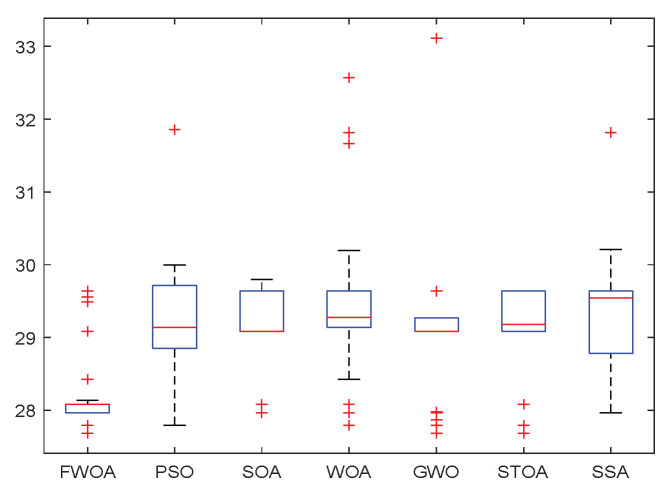
The Boxplot of Environment 5.

**Figure 17:**
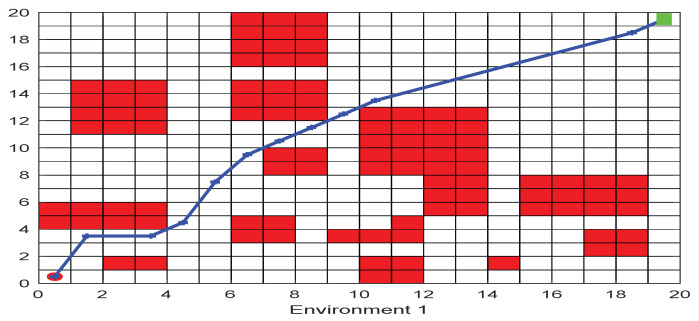
The Path of Environment 1.

**Figure 18:**
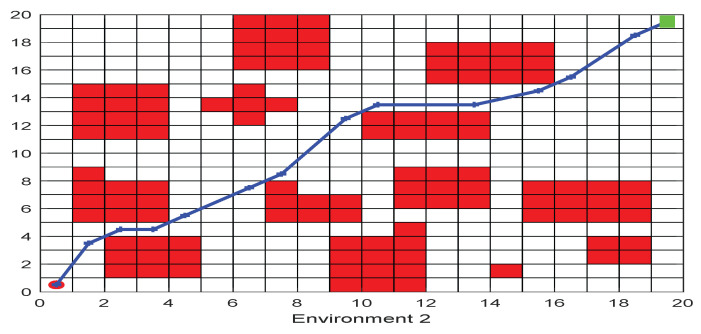
The Path of Environment 2.

**Figure 19:**
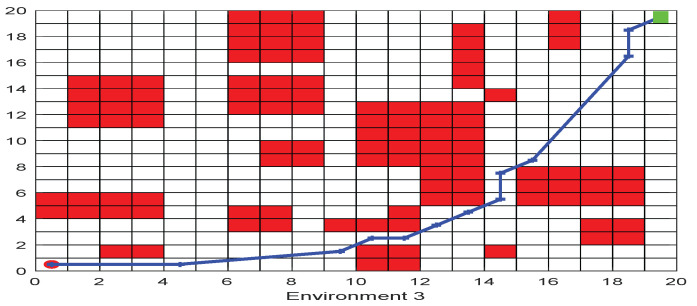
The Path of Environment 3.

**Figure 20:**
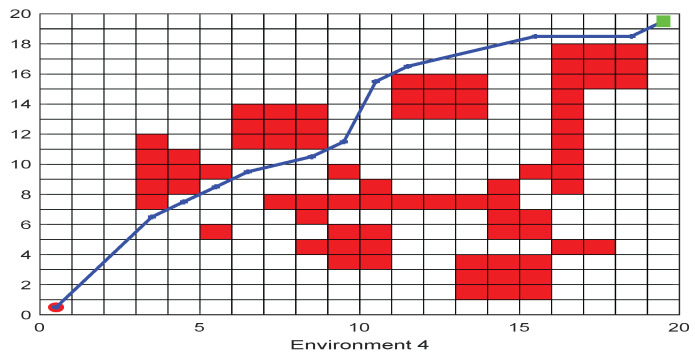
The Path of Environment 4.

**Figure 21:**
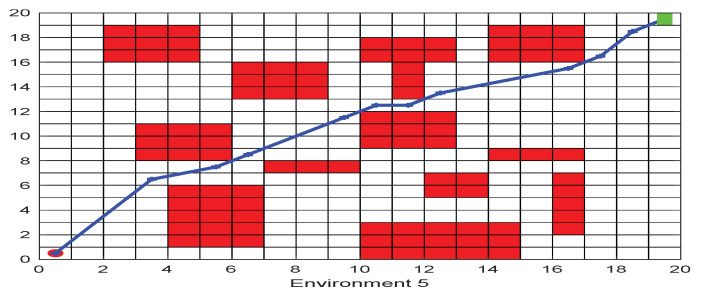
The Path of Environment 5.

As shown in Figure 6, Figure 7, Figure 8, Figure 9, Figure 10, Figure 11, Figure 12, Figure 13, Figure 14, Figure 15, Figure 16, Figure 17, Figure 18, Figure 19, Figure 20 and Figure 21, for the irregular obstacle environment with no influence range, five working environments are established: Environment 1, Environment 2, Environment 3, Environment 4, and Environment 5. The size of the map is set to 20 × 20, and the complexity of the map is gradual. The test is divided into three groups: (1) Environment 1 and Environment 2 are mainly used to test the existence of obstacles between the starting point and the map; (2) Environment 3 is used to test the existence of obstacles in the whole map; and (3) Environment 4 and Environment 5 are used to test the existence of obstacles in the middle of the map and near the target point. Complex maps are a challenge for mobile robots. Through the above tests, we can find the global optimal path and prove the excellent performance of FWOA. The starting point of the map is (0,0), represented by a red circle; the target point is (20,20), represented by a green square; and the outline of the obstacle is represented by a red rectangle. The number of iterations is set to 500, the number of population agents is set to 60, and the dimension is set to 30. The algorithm runs independently 30 times in each environment. Meanwhile, the algorithm is tested for a *p*-value to show the difference between the two algorithms.

**Figure 22:**
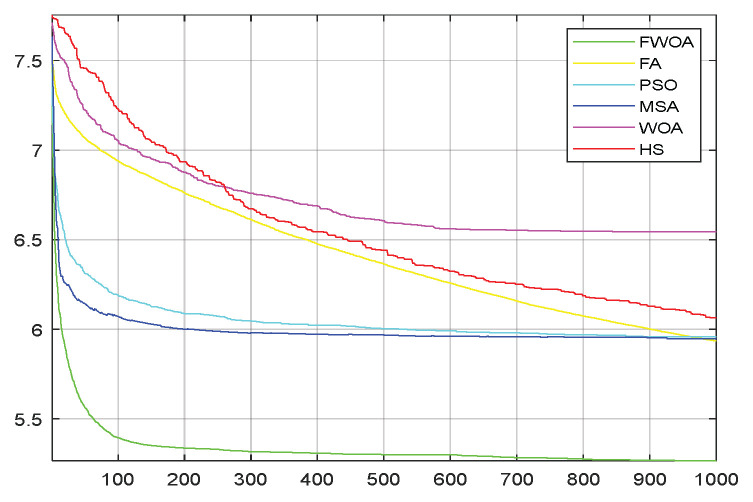
The convergence graph of environment 6.

**Figure 23:**
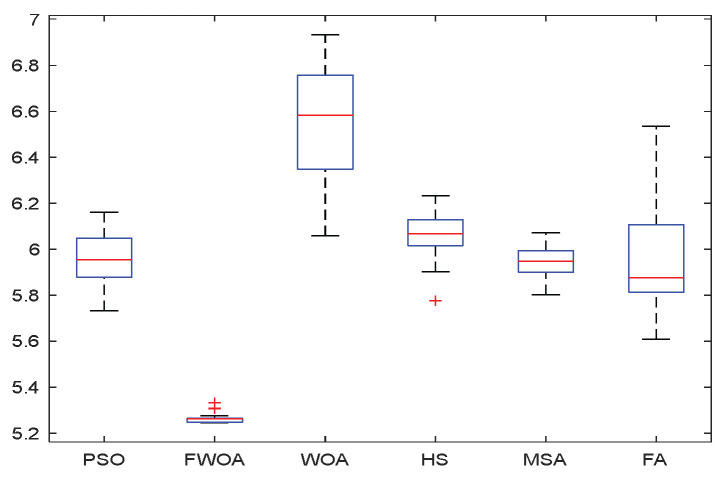
The Boxplot of Environment 6.

**Figure 24:**
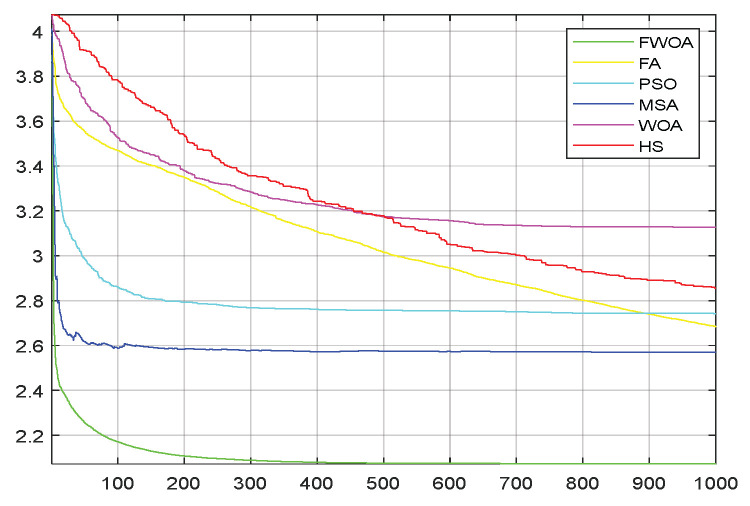
The convergence graph of environment 7.

**Figure 25:**
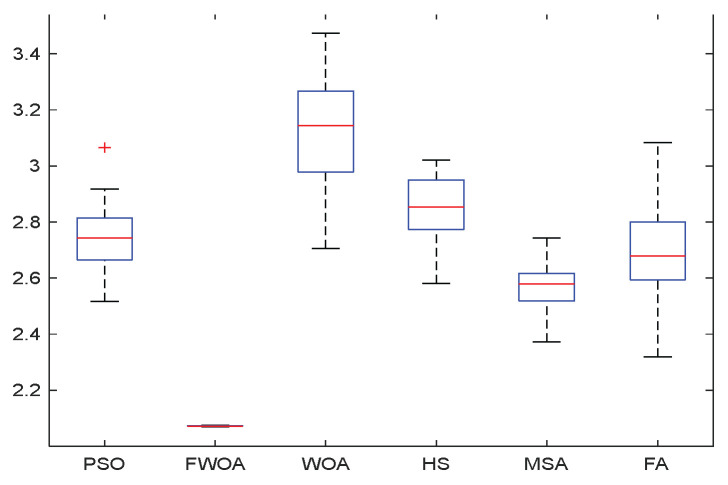
The Boxplot of Environment 7.

**Figure 26:**
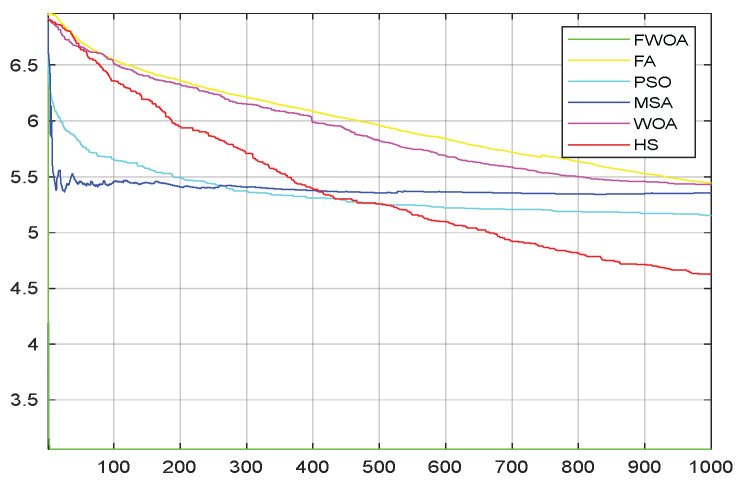
The convergence graph of environment 8.

**Figure 27:**
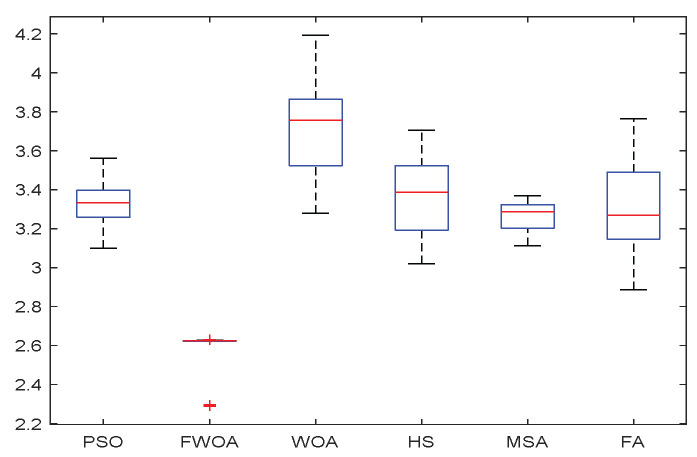
The Boxplot of Environment 8.

**Figure 28:**
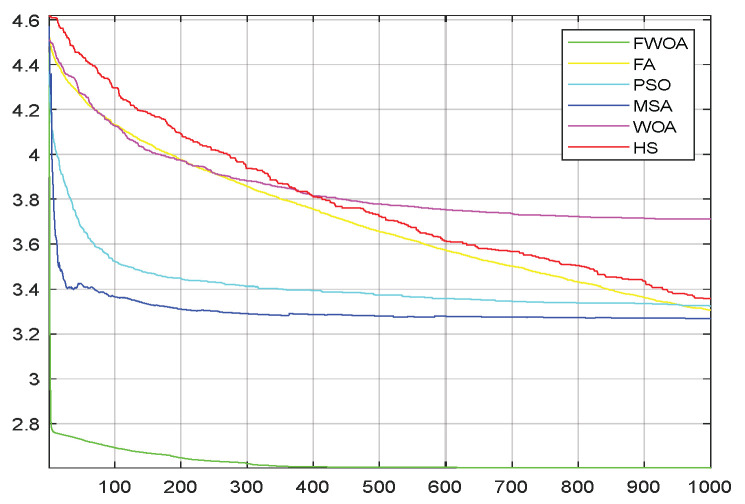
The convergence graph of environment 9.

**Figure 29:**
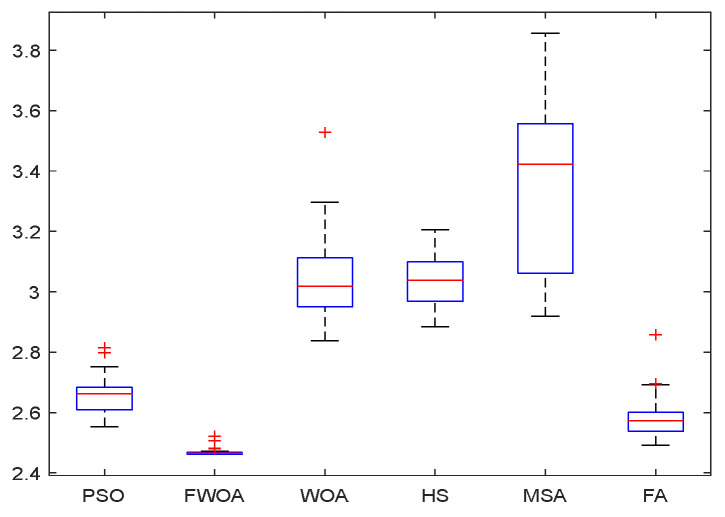
The Boxplot of Environment 9.

**Figure 30:**
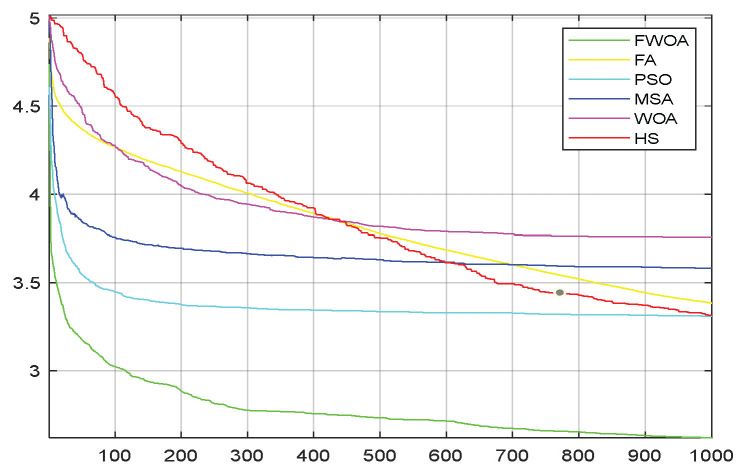
The convergence graph of environment 10.

**Figure 31:**
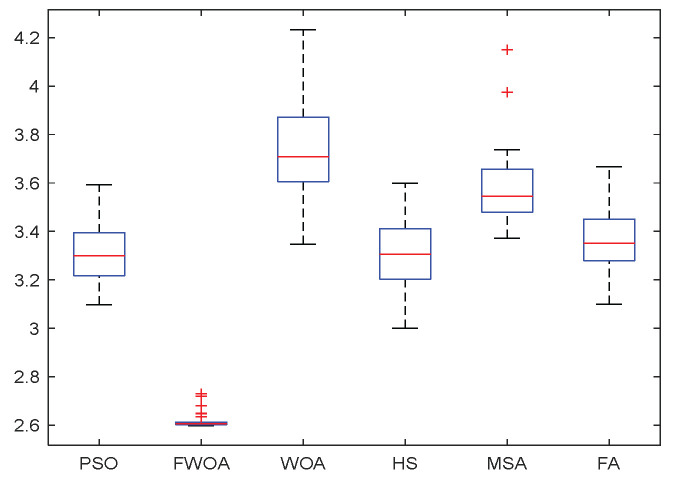
The Boxplot of Environment 10.

As shown in Figure 22, Figure 23, Figure 24, Figure 25, Figure 26, Figure 27, Figure 28, Figure 29, Figure 30 and Figure 31, five working environments are established for the regular obstacle environments with influence range: Environment 6, Environment 7, Environment 8, Environment 9, and Environment 10, with the influence range of circular. The starting point is (0,0) (represented by a black *), the target point is (500,0) (represented by a hollow square), and the obstacle is represented by a circle. The influence of obstacles decreases with an increase in radius. The number of iterations is set to 500, the dimension is set to 30, and the population number is set to 60. The algorithm runs independently 30 times in each environment. In order to show the difference between the algorithms, they are also tested in an experimental environment.

All simulations are implemented in MATLAB R2022a and run on an AMD Ryzen 9 5900HX with a Radeon Graphics CPU at 3.30 GHz and 16 GB of RAM under Windows 11.

### 6.2. Result Analysis

**Figure 32:**
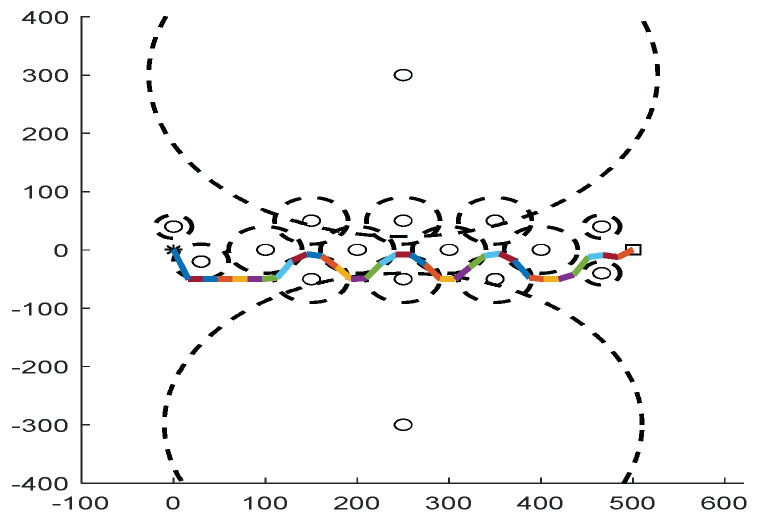
The Path of Environment 6.

**Figure 33:**
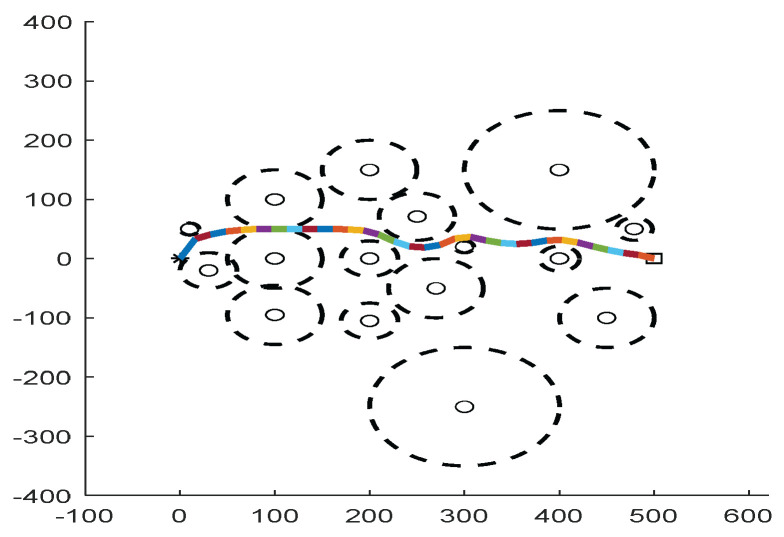
The Path of Environment 7.

**Figure 34:**
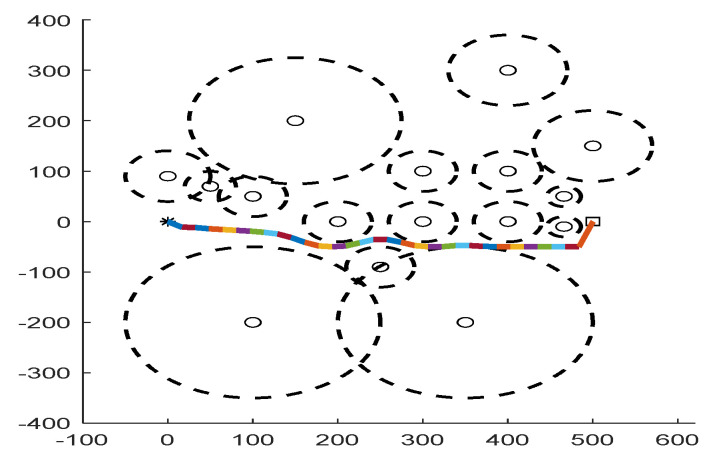
The Path of Environment 8.

**Figure 35:**
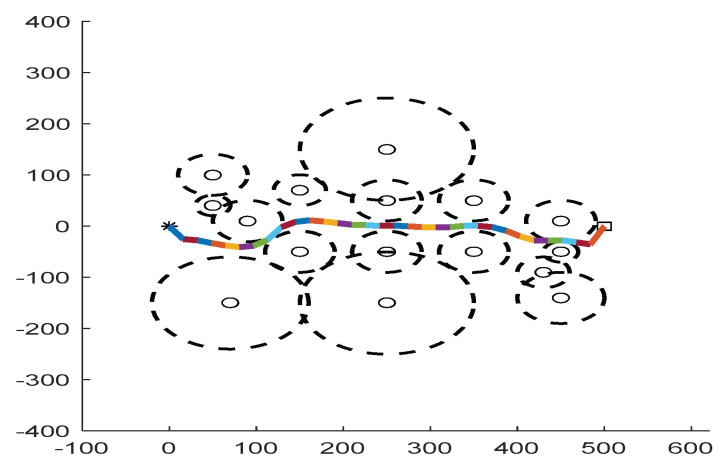
The Path of Environment 9.

**Figure 36:**
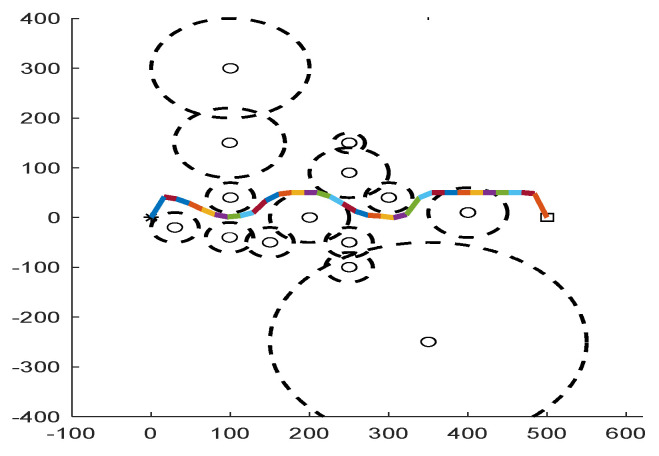
The Path of Environment 10.

This section analyzes the results of the experiments. The experiments are divided into two groups for analysis: (1) The experimental results of mobile robots in the irregular obstacle environment with no influence range are shown in Table 8, Table 9, Table 10, Table 11 and Table 12; the algorithm convergence graphs and algorithm box-plot graphs are shown in Figure 7, Figure 8, Figure 9, Figure 10, Figure 11, Figure 12, Figure 13, Figure 14, Figure 15 and Figure 16; the path graphs are shown in Figure 17, Figure 18, Figure 19, Figure 20 and Figure 21; and the *p*-value is shown in Table 13; (2) The experimental results of the mobile robot in the regular obstacle environments with influence range are shown in Table 14, Table 15, Table 16, Table 17 and Table 18; the algorithm convergence graphs and algorithm box-plot graphs are shown in Figure 22, Figure 23, Figure 24, Figure 25, Figure 26, Figure 27, Figure 28, Figure 29, Figure 30 and Figure 31; the path graphs are shown in Figure 32, Figure 33, Figure 34, Figure 35 and Figure 36; and the *p*-value is shown in Table 18. Through the analysis of ten working environments in two groups of experiments, we can know that FWOA has excellent performance and remarkable stability.

#### 6.2.1. Irregular Obstacles with None Influence Range

The selectable path of a robot is inversely proportional to the density of obstacles, and obstacles at different positions have different effects on path selection. As shown in Figure 17 and Figure 18, because of the limitation of the robot’s moving direction, the irregular obstacles near the starting point are the main influence on the path, which determines the subsequent moving direction of the robot. However, because of the path-planning method of FWOA, this influence is reduced. Meanwhile, because of the balance between exploitation and exploration, FWOA can always find the best moving path. Figure 19 shows the working environment of global obstacles. In this environment, due to the dense obstacles, robot path planning needs to focus on the judgment of path legitimacy. Figure 20 and Figure 21 show the obstacle environment near the target point, which is relatively simple compared with environments 1 and 3.

The experimental results are shown in Table 8, Table 9, Table 10, Table 11 and Table 12. For the path planning problem of mobile robots, Zhang Zhen proposed a new neighborhood search strategy to improve the fitness value of the global optimal individual. This paper found a search method based on the search population through the inspiration of Ref. [43]. From the experimental results, this method has a significant effect. The best mobile path can be found in each mobile robot’s working environment. Meanwhile, after 30 independent experiments in five working environments, the average moving path length of FWOA is the minimum, and the optimal value found is also among the best. It can be seen from the standard deviation that FWOA is also very stable and robust.

It can be seen from the convergence graphs that FWOA always converges the earliest among the six algorithms, which means that FWOA has a strong exploration ability and can quickly traverse the search space to find the optimal path. Compared with other algorithms, FWOA converges faster than them. This is due to the food-perception mechanism of the algorithm. While different populations of searchers search for the best, the perception of the search space (neighborhood) improves the exploration ability of the algorithm. Thus, the performance of FWOA is competitive in the algorithm. It can be seen from the standard deviation graphs that the stability of FWOA is not inferior to that of other algorithms and is on the same level as that of other algorithms. In general, the stability of FWOA is remarkable. It can be seen from Table 13 that FWOA is significantly different from other algorithms in the irregular obstacle environment without influence range and can maintain its independence.

To sum up, in the irregular obstacles with no influence range, FWOA has shown remarkable performance, and it is also very competitive.

#### 6.2.2. Regular Obstacle Environment with Influence Range

This section describes the performance of FWOA in a regular Obstacle environment with an influence range. In order to study the efficiency of different algorithms and show the performance of FWOA, this paper compares FWOA with classical Swarm Optimization (PSO) [20], Firefly algorithm (FA) [21], WOA [23], Seagull Optimization Algorithm (SOA) [33], Particle Sooty Tern Optimization Algorithm (STOA) [42], and Harmony Search (HS) [44].

From the convergence graphs of the algorithm (Figure 22, Figure 24, Figure 26, Figure 28 and Figure 30), we can know that the FWOA algorithm has a better convergence rate than other algorithms. Due to its good exploration capability, FWOA can not only converge rapidly but also ensure accuracy. Compared with the classical WOA, the exploration capability of the FWOA is almost twice that of the classical WOA. Thus, we can know that the performance of FWOA is very competitive.

From the boxplot (Figure 23, Figure 25, Figure 29 and Figure 31), it can be seen that the length of the optimal path found by FWOA after 30 independent operations changes very little, which means that FWOA has remarkable stability, and the optimal value can be found in each operation. Combined with the convergence graphs of the algorithm, FWOA has remarkable accuracy, stability, and robustness compared with other algorithms, which is attributed to the multi-population mechanism of the algorithm, which realizes the balance between exploitation and exploration.

It can be seen from the path graphs (Figure 32, Figure 33, Figure 34, Figure 35 and Figure 36) that the path found by FWOA avoids all the obstacle-affected areas to a certain extent, especially in areas with dense obstacles such as Environment 3. FWOA can avoid all the obstacle-affected areas and reach the target point, which indicates that FWOA has strong optimization ability.

The experimental results (Table 14, Table 15, Table 16, Table 17 and Table 18) show that FWOA has won first place in each experiment, and the path length is significantly smaller than other algorithms. Meanwhile, the worst case of the experimental results of FWOA is also better than the best case of other algorithms. It can be seen from Table 19 that FWOA is significantly different from other algorithms in the regular obstacle environment with influence range and can maintain its independence. The experimental results show that FWOA has remarkable performance.

In conclusion, FWOA can balance exploitation and exploration and has strong stability. It has demonstrated its competitiveness in experiments and has remarkable performance in solving practical problems, which can be applied to more complex practical problems.

## 7. Conclusions and Future Work

This paper intends to verify the performance of FWOA and its ability to deal with the MRPP by comparing it with other intelligent algorithms. In the MRPP, the traditional algorithm has the weakness of easily falling into local optima and exhibits slow convergence [45]. For the above reasons, this paper proposes FWOA to solve these problems. FWOA has the characteristics of fast convergence, remarkable exploration, and strong optimization ability. The algorithm is studied on 23 benchmark functions to analyze the exploitation, exploration, and convergence behavior of the algorithm, and WOA is found to be sufficiently competitive with other metaheuristic algorithms. Meanwhile, this paper experiments with the algorithm in two different environments and analyzes its ability to solve practical problems. The experiment results show that the algorithm has made significant progress, which indicates that FWOA has great advantages in solving MRPP. In the future, applying FWOA to complex, large-scale practical application problems will be meaningful.

## Figures and Tables

**Table 1:** Various MRPP solving methods.

Document	Method
[2]	collaborative path planning algorithm
[3]	static and dynamic environments
[6]	water flow potential field method and beetle antennae search algorithm
[7]	ant colony optimization
[9]	water flow potential field method and beetle antennae search algorithm
[11]	optimization and reinforcement learning
[12]	new approach based on Bezier curves
[13]	FIMOPSO
[16]	self-adaptive learning particle swarm optimization

**Table 2:** Uni-modal functions.

Function	Dimension	Range	*f*_min_
$f_{1}=\sum_{i=1}^{n}{x_{i}}^{2}$	30	[−100,100]	0
$f_{2}=\sum_{i=1}^{n}\left|x_{i}\right|+\prod_{i=1}^{n}\left|x_{i}\right|$	30	[−10,10]	0
$f_{3}=\sum_{i=1}^{n}(\sum_{j=1}^{i}{x_{j})}^{2}$	30	[−100,100]	0
$f_{4}=max\{\left|x_{i}\right|,1\leq i\leq n\}$	30	[−100,100]	0
$f_{5}=\sum_{i=1}^{n-1}\left[100{\left(x_{i+1}{{-x}_{i}}^{2}\right)}^{2}+{\left(x_{i}-1\right)}^{2}\right]$	30	[−30,30]	0
$f_{6}=\sum_{i=1}^{n}{{(x}_{i}+0.5)}^{2}$	30	[−100,100]	0
$f_{7}=\sum_{i=1}^{n}{{ix}_{i}}^{4}+Random\;[0,1)$	30	[−1.28,1.28]	0

**Table 3:** Multi-modal functions.

Function	Dimension	Range	*f*_min_
$f_{8}=\sum_{i=1}^{n}-x_{i}\sin\sqrt{\left|x_{i}\right|}$	30	[−500,500]	−418.9829×5
$f_{9}=\sum_{i=1}^{n}\left[{x_{i}}^{2}-10cos(2\pi x_{i})+10\right]$	30	[−5.12,5.12]	0
$f_{10}=-20exp(-0.2\sqrt{\frac{1}{n}\sum_{j=1}^{n}x_{j}})-exp(\frac{1}{n}cos(2\pi x_{j}))+20+e$	30	[−32,32]	0
$f_{11}=\frac{1}{4000}\sum_{i=1}^{n}{x_{i}}^{2}-\prod_{i=1}^{n}\cos\left(\frac{x_{i}}{\sqrt{i}}\right)+1$	30	[−600,600]	0
$f_{12}=\frac{\pi }{n}\left\{10\sin\left(\pi y_{1}\right)+\sum_{i=1}^{n-1}{\left(y_{i}-1\right)}^{2}\left[1+10\sin^{2}\left(\pi y_{i+1}\right)\right]+{\left(y_{i}-1\right)}^{2}\right\}+\sum_{i=1}^{n}u(x_{i},10,100,4)\;y_{i}=1+\frac{x_{i}+1}{4}\;u\left(x_{i},a,k,m\right)=\left\{\begin{array}{c} k{\left(x_{i}-a\right)}^{m}\;\;\;\;\;\;\;\;\;\;\;\;\;x_{i}>a\\ 0\;\;\;\;\;\;\;\;\;\;\;\;\;\;\;\;\;\;\;\;\;{-a<x}_{i}<a\\ k{\left(-x_{i}-a\right)}^{m}\;\;\;\;\;\;\;x_{i}<-a \end{array}\right.$	30	[−50,50]	0
$f_{13}=0.1\left\{10{sin}^{2}(3\pi x_{1})+\sum_{i=1}^{n}{\left(x_{i}-1\right)}^{2}[1+{sin}^{2}(3\pi x_{i}+1)]+{\left(x_{n}-1\right)}^{2}[1+{sin}^{2}(2\pi x_{n})]\right\}+\sum_{i=1}^{n}u(x_{i},5100.4)$	30	[−50,50]	0

**Table 4:** Fixed-dimension multi-modal functions.

Function	Dimension	Range	*f*_min_
$f_{14}={\left(\frac{1}{500}+\sum_{j=1}^{25}\frac{1}{j+\sum_{i=1}^{2}{\left(x_{i}-a_{ij}\right)}^{6}}\right)}^{-1}$	2	[−65,65]	1
${\mathrm{sf}}_{15}=\sum_{i=1}^{11}{\left[a_{i}-\frac{x_{1}({b_{i}}^{2}+b_{i}x_{2})}{{b_{i}}^{2}+b_{i}x_{3}+x_{4}}\right]}^{2}$	4	[−5,5]	0.00030
$f_{16}=4x_{1}^{2}-2.1x_{1}^{4}+\frac{1}{3}x_{1}^{6}+x_{1}x_{2}-4x_{2}^{2}+4x_{2}^{4}$	2	[−5,5]	−1.0316
$f_{17}={{(x}_{2}-\frac{5.1}{{4\pi }^{2}}x_{1}^{2}+\frac{5}{\pi }x_{1}-6)}^{2}+10\left(1-\frac{1}{8\pi }\right)cosx_{1}+10$	2	[−5,5]	0.398
$f_{18}=\left[1+{\left(x_{1}+x_{2}+1\right)}^{2}\left(19-14x_{1}+3x_{1}^{2}-14x_{2}+6x_{1}x_{2}+3x_{2}^{2}\right)\right]\times [30+{\left(2x_{1}-3x_{2}\right)}^{2}\times (18-32x_{1}+12x_{1}^{2}+48x_{2}-36x_{1}x_{2}+27x_{2}^{2})]$	2	[−2,2]	3
$f_{19}=-\sum_{i=1}^{4}c_{i}exp(-\sum_{j=1}^{3}a_{ij}{\left(x_{j}-p_{ij}\right)}^{2})$	3	[1,3]	−3.86
$f_{20}=-\sum_{i=1}^{4}c_{i}exp(-\sum_{j=1}^{6}a_{ij}{\left(x_{j}-p_{ij}\right)}^{2})$	6	[0,1]	−3.32
$f_{21}=-\sum_{i=1}^{5}{\left[(X-a_{i}){{\left(X-a_{i}\right)}^{T}+c}_{i})\right]}^{-1}$	4	[0,10]	−10.1532
$f_{22}=-\sum_{i=1}^{7}{\left[(X-a_{i}){{\left(X-a_{i}\right)}^{T}+c}_{i})\right]}^{-1}$	4	[0,10]	−10.4028
$f_{23}=-\sum_{i=1}^{10}{\left[(X-a_{i}){{\left(X-a_{i}\right)}^{T}+c}_{i})\right]}^{-1}$	4	[0,10]	−10.4028

**Table 5:** The result of Uni-modal functions.

		FWOA	WOA	SOA	PSO	GWO	STOA	SSA
$f_{1}$	Min	0	8.430261 × 10^−210^	1.555097 × 10^−36^	2.810415 × 10^−84^	6.393086 × 10^−89^	1.635800 × 10^−23^	4.769387 × 10^−09^
	Max	5.273753 × 10^−171^	7.652315 × 10^−32^	7.652315 × 10^−32^	4.526335 × 10^−76^	1.821216 × 10^−84^	2.765344 × 10^−20^	9.360340 × 10^−09^
	Ave	2.854804 × 10^−172^	9.126593 × 10^194^	3.559156 × 10^−33^	4.031582 × 10^−77^	2.397316 × 10^−85^	4.012824 × 10^−21^	7.021318 × 10^−09^
	Std.	0	1.956124 × 10^−64^	1.956124 × 10^−64^	1.292897 × 10^−152^	2.043497 × 10^−169^	4.681402 × 10^−41^	1.419605 × 10^−18^
$f_{2}$	Min	0	3.336010 × 10^−123^	1.228525 × 10^−21^	1.047446 × 10^−12^	1.465409 × 10^−50^	2.186051 × 10^−15^	4.064451 × 10^−05^
	Max	9.873765 × 10^−104^	5.406626 × 10^−20^	5.406626 × 10^−20^	3.829112 × 10^−06^	2.796488 × 10^−48^	4.621727 × 10^−13^	2.059145 × 10^+00^
	Ave	5.485366 × 10^−105^	5.434907 × 10^−112^	1.048486 × 10^−20^	2.593201 × 10^−07^	3.096340 × 10^−49^	8.820698 × 10^−14^	2.282708 × 10^−01^
	Std.	4.020400 × 10^−208^	1.286922 × 10^−40^	1.286922 × 10^−40^	5.202686 × 10^−13^	2.706440 × 10^−97^	1.466744 × 10^−26^	2.252014 × 10^−01^
$f_{3}$	Min	0	4.745790 × 10^+01^	1.042476 × 10^−23^	2.851281 × 10^−04^	3.116347 × 10^−32^	5.942626 × 10^−14^	5.760246 × 10^−02^
	Max	5.961890 × 10^+00^	3.403934 × 10^−17^	3.403934 × 10^−17^	8.574375 × 10^−03^	4.108705 × 10^−24^	3.588812 × 10^−10^	8.382606 × 10^+00^
	Ave	2.101297 × 10^−01^	4.010754 × 10^+03^	2.264491 × 10^−18^	2.048678 × 10^−03^	1.416761 × 10^−25^	2.915104 × 10^−11^	8.969260 × 10^−01^
	Std.	1.181940 × 10^+00^	5.174733 × 10^−35^	5.174733 × 10^−35^	3.242489 × 10^−06^	5.616559 × 10^−49^	5.133289 × 10^−21^	2.658013 × 10^+00^
$f_{4}$	Min	0	4.299918 × 10^−07^	4.505221 × 10^−13^	1.229180 × 10^−03^	4.800334 × 10^−23^	1.128292 × 10^−07^	4.350890 × 10^−04^
	Max	1.117087 × 10^−03^	3.594542 × 10^−08^	3.594542 × 10^−08^	2.791385 × 10^−02^	1.432535 × 10^−20^	4.542304 × 10^−06^	4.277223 × 10^+00^
	Ave	3.777352 × 10^−05^	1.715458 × 10^+01^	1.323292 × 10^−09^	7.525940 × 10^−03^	1.617966 × 10^−21^	6.961723 × 10^−07^	5.554818 × 10^−01^
	Std.	4.155907 × 10^−08^	4.291170 × 10^−17^	4.291170 × 10^−17^	3.887478 × 10^−05^	8.220771 × 10^−42^	7.019692 × 10^−13^	6.972356 × 10^−01^
$f_{5}$	Min	1.660556 × 10^−02^	2.544580 × 10^+01^	2.596043 × 10^+01^	3.548720 × 10^−01^	2.487610 × 10^+01^	2.620052 × 10^+01^	2.152424 × 10^+01^
	Max	2.695590 × 10^+01^	2.861168 × 10^+01^	2.861168 × 10^+01^	7.741710 × 10^+01^	2.712737 × 10^+01^	2.873763 × 10^+01^	5.826600 × 10^+02^
	Ave	2.050639 × 10^+01^	2.591175 × 10^+01^	2.753575 × 10^+01^	4.015986 × 10^+01^	2.633584 × 10^+01^	2.751388 × 10^+01^	9.092186 × 10^+01^
	Std.	1.082212 × 10^+02^	3.846561 × 10^−01^	3.846561 × 10^−01^	7.837989 × 10^+02^	4.753373 × 10^−01^	4.483316 × 10^−01^	1.561153 × 10^+04^
$f_{6}$	Min	1.306296 × 10^−04^	1.480551 × 10^−04^	1.633545 × 10^+00^	0	5.460670 × 10^−06^	7.128582 × 10^−01^	3.853132 × 10^−09^
	Max	5.012690 × 10^−04^	3.248522 × 10^+00^	3.248522 × 10^+00^	2.899680 × 10^−29^	5.060667 × 10^−01^	2.508231 × 10^+00^	8.553629 × 10^−09^
	Ave	3.071433 × 10^−04^	3.234442 × 10^−04^	2.462839 × 10^+00^	1.416252 × 10^−30^	1.159188 × 10^−01^	1.500161 × 10^+00^	6.730791 × 10^−09^
	Std.	8.797922 × 10^−09^	1.861930 × 10^−01^	1.861930 × 10^−01^	2.971108 × 10^−59^	2.452040 × 10^−02^	1.996132 × 10^−01^	1.571142 × 10^−18^
$f_{7}$	Min	9.352327 × 10^−07^	2.738278 × 10^−05^	3.030240 × 10^−05^	2.342989 × 10^−03^	8.511159 × 10^−05^	1.283198 × 10^−04^	9.841093 × 10^−03^
	Max	8.370940 × 10^−04^	6.483309 × 10^−04^	6.483309 × 10^−04^	7.825572 × 10^−03^	4.966548 × 10^−04^	3.612736 × 10^−03^	5.114226 × 10^−02^
	Ave	1.123975 × 10^−04^	5.837796 × 10^−04^	2.616332 × 10^−04^	5.040257 × 10^−03^	2.489494 × 10^−04^	8.743143 × 10^−04^	2.689375 × 10^−02^
	Std.	4.441664 × 10^−08^	3.626657 × 10^−08^	3.626657 × 10^−08^	2.025908 × 10^−06^	1.153306 × 10^−08^	5.463082 × 10^−07^	1.042387 × 10^−04^

**Table 6:** The result of Multi-modal functions.

		FWOA	WOA	SOA	PSO	GWO	STOA	SSA
$f_{8}$	Min	−1.256946 × 10^+04^	−1.256945 × 10^+04^	−7.887318 × 10^+03^	−8.423960 × 10^+03^	−7.457098 × 10^+03^	−7.580349 × 10^+03^	−9.210553 × 10^+03^
	Max	−9.862899 × 10^+03^	−5.030709 × 10^+03^	−5.030709 × 10^+03^	−5.561062 × 10^+03^	−3.605024 × 10^+03^	−5.132615 × 10^+03^	−5.633845 × 10^+03^
	Ave	−1.208077 × 10^+04^	−1.179017 × 10^+04^	−6.157293 × 10^+03^	−6.743667 × 10^+03^	−6.337316 × 10^+03^	−5.889671 × 10^+03^	−7.647054 × 10^+03^
	Std.	5.006503 × 10^+05^	6.502353 × 10^+05^	6.502353 × 10^+05^;	5.045922 × 10^+05^	6.460060 × 10^+05^	3.280557 × 10^+05^	9.304187 × 10^+05^
$f_{9}$	Min	0	0	0	2.089413 × 10^+01^	0	0	1.492438 × 10^+01^
	Max	0	5.684342 × 10^−14^	5.684342 × 10^−14^	8.457133 × 10^+01^	5.684342 × 10^−14^	1.260584 × 10^+01^	9.949549 × 10^+01^
	Ave	0	3.789561 × 10^−15^	1.894781 × 10^−15^	4.424245 × 10^+01^	3.789561 × 10^−15^	7.857429 × 10^−01^	4.092592 × 10^+01^
	Std.	0	1.077058 × 10^−28^	1.077058 × 10^−28^	2.612571 × 10^+02^	2.079836 × 10^−28^	6.245225 × 10^+00^	3.038580 × 10^+02^
$f_{10}$	Min	8.881784 × 10^−16^	8.881784 × 10^−16^	1.509903 × 10^−14^	7.993606 × 10^−15^	7.993606 × 10^−15^	1.995507 × 10^+01^	1.575264 × 10^−05^
	Max	4.440892 × 10^−15^	1.996086 × 10^+01^	1.996086 × 10^+01^	1.899744 × 10^+00^	1.509903 × 10^−14^	1.995985 × 10^+01^	3.158812 × 10^+00^
	Ave	1.125026 × 10^−15^	4.440892 × 10^−15^	1.929332 × 10^+01^	3.073540 × 10^−01^	1.036208 × 10^−14^	1.995836 × 10^+01^	1.237668 × 10^+00^
	Std.	8.124361 × 10^−31^	1.327821 × 10^+01^	1.327821 × 10^+01^	3.497835 × 10^−01^	8.994828 × 10^−30^	1.435757 × 10^−06^	1.132933 × 10^+00^
$f_{11}$	Min	0	0	0	0	0	0	3.676968 × 10^−02^
	Max	0	2.077809 × 10^−02^	2.077809 × 10^−02^	4.672941 × 10^−02^	2.022412 × 10^−02^	8.989056 × 10^−02^	1.286951 × 10^−08^
	Ave	0	8.562736 × 10^−04^	6.926030 × 10^−04^	9.768366 × 10^−03^	9.371244 × 10^−04^	8.318053 × 10^−03^	7.221352 × 10^−03^
	Std.	0	1.439097 × 10^−05^	1.439097 × 10^−05^;	1.087677 × 10^−04^	1.534190 × 10^−05^	4.163995 × 10^−04^	7.826887 × 10^−05^
$f_{13}$	Min	2.108763 × 10^−05^	2.380328 × 10^−05^	1.107146 × 10^−01^	1.578612 × 10^−32^	4.409217 × 10^−07^	5.906150 × 10^−02^	2.240856 × 10^−11^
	Max	8.51455 7 × 10^−05^	3.001715 × 10^−01^	3.001715 × 10^−01^	5.182541 × 10^−01^	3.571543 × 10^−02^	2.162930 × 10^−01^	5.905217 × 10^+00^
	Ave	4.197168 × 10^−05^	4.948684 × 10^−04^	1.978382 × 10^−01^	4.491879 × 10^−02^	1.304051 × 10^−02^	1.144425 × 10^−01^	1.942916 × 10^+00^
	Std.	2.311110 × 10^−10^	3.789442 × 10^−03^	3.789442 × 10^−03^	1.162089 × 10^−02^	5.920125 × 10^−05^	2.306902 × 10^−03^	2.686687 × 10^+00^
$f_{14}$	Min	2.905554 × 10^−04^	3.394113 × 10^−04^	1.178007 × 10^+00^	1.473043 × 10^−32^	6.291644 × 10^−06^	6.834331 × 10^−01^	2.353540 × 10^−10^
	Max	2.905554 × 10^−04^	2.114193 × 10^+00^	2.114193 × 10^+00^	9.737116 × 10^−02^	4.123093 × 10^−01^	1.969527 × 10^+00^	4.394886 × 10^−02^
	Ave	4.052124 × 10^−03^	3.628557 × 10^−03^	1.677016 × 10^+00^	6.510216 × 10^−03^	1.609783 × 10^−01^	1.293502 × 10^+00^	6.560701 × 10^−03^
	Std.	1.334761 × 10^−02^	4.883671 × 10^−02^	4.883671 × 10^−02^	3.274727 × 10^−04^	1.209685 × 10^−02^	6.628387 × 10^−02^	8.727186 × 10^−05^

**Table 7:** The result of Fixed-dimension multi-modal functions.

		FWOA	WOA	SOA	PSO	GWO	STOA	SSA
$f_{14}$	Min	9.980038 × 10^−01^	9.980038 × 10^−01^	9.980038 × 10^−01^	9.980038 × 10^−01^	9.980038 × 10^−01^	9.980038 × 10^−01^	9.980038 × 10^−01^
	Max	9.980038 × 10^−01^	2.982105 × 10^+00^	2.982105 × 10^+00^	1.992031 × 10^+00^	1.076318 × 10^+01^	9.980038 × 10^−01^	9.980038 × 10^−01^
	Ave	9.980038 × 10^−01^	1.064141 × 10^+00^	1.064141 × 10^+00^	1.229943 × 10^+00^	2.117150 × 10^+00^	9.980038 × 10^−01^	9.980038 × 10^−01^
	Std.	1.166008 × 10^−23^	1.312219 × 10^−01^	1.312219 × 10^−01^	1.828534 × 10^−01^	3.621514 × 10^+00^	2.062775 × 10^−18^	3.995308 × 10^−32^
$f_{15}$	Min	3.074875 × 10^−04^	3.075390 × 10^−04^	3.076348 × 10^−04^	3.074860 × 10^−04^	3.074864 × 10^−04^	3.078072 × 10^−04^	3.074860 × 10^−04^
	Max	1.223604 × 10^−03^	1.256914 × 10^−03^	1.256914 × 10^−03^	1.594050 × 10^−03^	2.036334 × 10^−02^	1.595878 × 10^−03^	1.594901 × 10^−03^
	Ave	3.417386 × 10^−04^	5.549406 × 10^−04^	1.194463 × 10^−03^	4.361424 × 10^−04^	2.343596 × 10^−03^	1.205608 × 10^−03^	9.405784 × 10^−04^
	Std.	2.796709 × 10^−08^	2.809383 × 10^−08^	2.809383 × 10^−08^	1.541092 × 10^−07^	3.735096 × 10^−05^	3.336449 × 10^−08^	1.403797 × 10^−07^
$f_{16}$	Min	−1.031628 × 10^+00^	−1.031628 × 10^+00^	−1.031628 × 10^+00^	−1.031628 × 10^+00^	−1.031628 × 10^+00^	−1.031628 × 10^+00^	−1.031628 × 10^+00^
	Max	−1.031628 × 10^+00^	−1.031628 × 10^+00^	−1.031628 × 10^+00^	−1.031628 × 10^+00^	−1.031628 × 10^+00^	−1.031627 × 10^+00^	−1.031628 × 10^+00^
	Ave	−1.031628 × 10^+00^	−1.031628 × 10^+00^	−1.031628 × 10^+00^	−1.031628 × 10^+00^	−1.031628 × 10^+00^	−1.031628 × 10^+00^	−1.031628 × 10^+00^
	Std.	1.046047 × 10^−21^	1.456520 × 10^−14^	1.456520 × 10^−14^	4.590354 × 10^−31^	1.514620 × 10^−18^	6.415465 × 10^−14^	8.614565 × 10^−30^
$f_{17}$	Min	3.978874 × 10^−01^	3.978874 × 10^−01^	3.978877 × 10^−01^	3.978874 × 10^−01^	3.978874 × 10^−01^	3.978877 × 10^−01^	3.978874 × 10^−01^
	Max	3.978878 × 10^−01^	3.980685 × 10^−01^	3.980685 × 10^−01^	3.978874 × 10^−01^	3.978884 × 10^−01^	3.980266 × 10^−01^	3.978874 × 10^−01^
	Ave	3.978874 × 10^−01^	3.978874 × 10^−01^	3.979114 × 10^−01^	3.978874 × 10^−01^	3.978875 × 10^−01^	3.979088 × 10^−01^	3.978874 × 10^−01^
	Std.	8.514675 × 10^−15^	1.349701 × 10^−09^	1.349701 × 10^−09^	0.000000 × 10^+00^	3.859782 × 10^−14^	9.203009 × 10^−10^	1.178905 × 10^−28^
$f_{18}$	Min	3.000000 × 10^+00^	3.000000 × 10^+00^	3.000000 × 10^+00^	3.000000 × 10^+00^	3.000000 × 10^+00^	3.000000 × 10^+00^	3.000000 × 10^+00^
	Max	3.000004 × 10^+00^	3.000005 × 10^+00^	3.000005 × 10^+00^	3.000000 × 10^+00^	3.000008 × 10^+00^	3.000020 × 10^+00^	3.000000 × 10^+00^
	Ave	3.000000 × 10^+00^	3.000000 × 10^+00^	3.000001 × 10^+00^	3.000000 × 10^+00^	3.000001 × 10^+00^	3.000002 × 10^+00^	3.000000 × 10^+00^
	Std.	5.635206 × 10^−13^	2.210026 × 10^−12^	2.210026 × 10^−12^	1.740934 × 10^−30^	2.567861 × 10^−12^	1.625908 × 10^−11^	8.357709 × 10^−28^
$f_{19}$	Min	−3.862782 × 10^+00^	−3.862782 × 10^+00^	−3.862767 × 10^+00^	−3.862782 × 10^+00^	−3.862782 × 10^+00^	−3.862773 × 10^+00^	−3.862782 × 10^+00^
	Max	−3.862762 × 10^+00^	−3.854857 × 10^+00^	−3.854857 × 10^+00^	−3.862782 × 10^+00^	−3.856489 × 10^+00^	−3.854856 × 10^+00^	−3.862782 × 10^+00^
	Ave	−3.862778 × 10^+00^	−3.862627 × 10^+00^	−3.855427 × 10^+00^	−3.862782 × 10^+00^	−3.862571 × 10^+00^	−3.855671 × 10^+00^	−3.862782 × 10^+00^
	Std.	2.805649 × 10^−11^	3.958128 × 10^−06^	3.958128 × 10^−06^	7.344567 × 10^−30^	1.319696 × 10^−06^	5.734189 × 10^−06^	2.305378 × 10^−29^
$f_{20}$	Min	−3.321995 × 10^+00^	−3.321993 × 10^+00^	−3.200659 × 10^+00^	−3.321995 × 10^+00^	−3.321994 × 10^+00^	−3.321919 × 10^+00^	−3.321995 × 10^+00^
	Max	−3.321938 × 10^+00^	−2.840363 × 10^+00^	−2.840363 × 10^+00^	−3.203102 × 10^+00^	−3.134100 × 10^+00^	−3.015514 × 10^+00^	−3.202625 × 10^+00^
	Ave	−3.321976 × 10^+00^	−3.257065 × 10^+00^	−3.056846 × 10^+00^	−3.262549 × 10^+00^	−3.249134 × 10^+00^	−3.069592 × 10^+00^	−3.214881 × 10^+00^
	Std.	2.390144 × 10^−10^	5.765841 × 10^−03^	5.765841 × 10^−03^	3.655752 × 10^−03^	4.467225 × 10^−03^	5.893752 × 10^−03^	1.318810 × 10^−03^
$f_{21}$	Min	−1.015320 × 10^+01^	−1.015320 × 10^+01^	−1.014653 × 10^+01^	−1.015320 × 10^+01^	−1.015317 × 10^+01^	−1.014820 × 10^+01^	−1.015320 × 10^+01^
	Max	−1.015288 × 10^+01^	−4.965276 × 10^−01^	−4.965276 × 10^−01^	−2.630472 × 10^+00^	−5.100549 × 10^+00^	−4.982139 × 10^−01^	−5.055198 × 10^+00^
	Ave	−1.015312 × 10^+01^	−1.015317 × 10^+01^	−5.785824 × 10^+00^	−5.968921 × 10^+00^	−9.984578 × 10^+00^	−5.944664 × 10^+00^	−8.971262 × 10^+00^
	Std.	5.716151 × 10^−09^	1.600742 × 10^+01^	1.600742 × 10^+01^	1.007418 × 10^+01^	8.509064 × 10^−01^	1.798099 × 10^+01^	4.748450 × 10^+00^
$f_{22}$	Min	−1.040294 × 10^+01^	−1.040294 × 10^+01^	−1.039981 × 10^+01^	−1.040294 × 10^+01^	−1.040287 × 10^+01^	−1.039889 × 10^+01^	−1.040294 × 10^+01^
	Max	−1.040269 × 10^+01^	−9.080722 × 10^−01^	−9.080722 × 10^−01^	−1.837593 × 10^+00^	−1.040245 × 10^+01^	−9.080713 × 10^−01^	−5.087672 × 10^+00^
	Ave	−1.040288 × 10^+01^	−9.516997 × 10^+00^	−7.709851 × 10^+00^	−8.028456 × 10^+00^	−1.040271 × 10^+01^	−8.729593 × 10^+00^	−1.022576 × 10^+01^
	Std.	3.938498 × 10^−09^	1.272529 × 10^+01^	1.272529×e^+01^	1.219797 × 10^+01^	1.193802 × 10^−08^	1.032487 × 10^+01^	9.417361 × 10^−01^
$f_{23}$	Min	−1.053641 × 10^+01^	−1.053641 × 10^+01^	−1.053479 × 10^+01^	−1.053641 × 10^+01^	−1.053639 × 10^+01^	−1.053488 × 10^+01^	−1.053641 × 10^+01^
	Max	−1.053611 × 10^+01^	−9.488805 × 10^−01^	−9.488805 × 10^−01^	−2.421734 × 10^+00^	−2.421726 × 10^+00^	−9.488816 × 10^−01^	−5.175647 × 10^+00^
	Ave	−1.053635 × 10^+01^	−1.010588 × 10^+01^	−9.842207 × 10^+00^	−8.675884 × 10^+00^	−1.026572 × 10^+01^	−9.480819 × 10^+00^	−9.821641 × 10^+00^
	Std.	3.456077 × 10^−09^	4.688671 × 10^+00^	4.688671 × 10^+00^	1.028709 × 10^+01^	2.194825 × 10^+00^	6.051566 × 10^+00^	3.435321 × 10^+00^

**Table 8:** The result of environment 1.

	FWOA	WOA	PSO	GWO	STOA	SSA	SOA
Mean	28.4296	32.5538	31.0256	30.3024	30.4791	30.0335	30.3244
Best	27.5602	28.1236	27.7897	27.7897	27.7897	27.5602	27.5602
Worst	29.6515	48.1842	50.0459	34.3996	31.0189	31.0822	31.0189
Std.	0.579903	4.10306	5.23552	1.57175	0.920949	1.09488	0.948411

**Table 9:** The result of environment 2.

	FWOA	WOA	PSO	GWO	STOA	SSA	SOA
Mean	29.372	45.4813	31.6773	30.5589	29.4531	29.8522	29.458
Best	28.464	28.7003	28.464	28.8269	29.4046	28.8269	28.8269
worst	30.8352	400	51.6065	39.563	29.9166	30.8587	30.3269
Std.	0.562282	67.0978	4.60077	2.40512	0.134563	0.36874	0.224772

**Table 10:** The result of environment 3.

	FWOA	WOA	PSO	GWO	STOA	SSA	SOA
Mean	30.5562	45.7169	44.5951	31.0557	31.1485	31.3353	31.1643
Best	28.4268	29.8026	29.91	29.2198	30.8733	29.117	30.5405
Worst	34.6255	400	400	34.3996	31.575	32.1488	31.575
Std.	1.36528	66.9532	67.1539	0.923568	0.173375	0.542579	0.211504

**Table 11:** The result of environment 4.

	FWOA	WOA	PSO	GWO	STOA	SSA	SOA
Mean	28.7738	29.6893	29.5183	29.2697	28.8092	29.015	28.8804
Best	28.3121	28.7729	28.5277	28.3121	28.4902	28.6611	28.4902
Worst	29.0248	35.6569	34.439	32.8926	28.9801	29.5592	29.3811
Std.	0.1384	1.80256	1.36026	1.27619	0.121089	0.204619	0.164828

**Table 12:** The result of environment 5.

	FWOA	WOA	PSO	GWO	STOA	SSA	SOA
Mean	28.2203	29.4382	29.2153	29.0043	29.1479	29.2951	29.2025
Best	27.6813	27.7949	27.7949	27.6813	27.6813	27.9662	27.9662
Worst	29.6366	32.5697	31.8578	33.1142	29.6366	31.8174	29.7949
Std.	0.512914	1.09052	0.807675	0.972006	0.577413	0.793053	0.472232

**Table 13:** The *p*-value of experiments.

	Environment 1	Environment 2	Environment 3	Environment 4	Environment 5
WOA vs. FWOA	3.54595 × 10^−154^	1.23598 × 10^−158^	1.04784 × 10^−154^	1.09486 × 10^−161^	8.03319 × 10^−99^
PSO vs. FWOA	1.12188 × 10^−107^	1.06873 × 10^−71^	8.32723 × 10^−30^	7.92567 × 10^−154^	1.29799 × 10^−54^
GWO vs. FWOA	7.60116 × 10^−127^	1.25814 × 10^−104^	2.91533 × 10^−154^	4.87965 × 10^−158^	4.75254 × 10^−60^
STOA vs. FWOA	1.72411 × 10^−123^	4.22364 × 10^−13^	1.02367 × 10^−50^	2.64802 × 10^−79^	5.51899 × 10^−98^
SSA vs. FWOA	2.55839 × 10^−105^	4.28541 × 10^−19^	1.29613 × 10^−52^	1.63432 × 10^−138^	6.13455 × 10^−82^
SOA vs. FWOA	6.62596 × 10^−126^	3.5665 × 10^−20^	2.33206 × 10^−57^	6.97704 × 10^−122^	1.83984 × 10^−107^

**Table 14:** The result of environment 6.

	FWOA	PSO	WOA	HS	FA	MSA
Mean	5.2656	5.95772	6.54454	6.06448	5.93502	5.94622
Best	5.24577	5.73289	6.05951	5.77577	5.60869	5.80207
Worst	5.33313	6.16154	6.93088	6.23352	6.53488	6.07259
Std.	0.000463621	0.0139897	0.0595565	0.00850826	0.0418203	0.00479293

**Table 15:** The result of environment 7.

	FWOA	PSO	WOA	HS	FA	MSA
Mean	2.07199	2.74251	3.12788	2.85464	2.68428	2.5689
Best	2.07047	2.51704	2.70531	2.58145	2.31947	2.37212
Worst	2.07512	3.06511	3.47429	3.02096	3.08297	2.74311
Std.	1.26108 × 10^−06^	0.012969	0.0389334	0.0117401	0.0274849	0.00630382

**Table 16:** The result of environment 8.

	FWOA	PSO	WOA	HS	FA	MSA
Mean	2.60248	3.32557	3.71071	3.35789	3.30654	3.27013
Best	2.28948	3.09882	3.27964	3.01973	2.88651	3.11258
Worst	2.62992	3.5623	4.19325	3.70561	3.76308	3.36859
Std.	0.00709538	0.013238	0.0484691	0.0347663	0.0423018	0.00482256

**Table 17:** The result of environment 9.

	FWOA	PSO	WOA	HS	FA	MSA
Mean	2.46952	2.66323	3.04348	3.03596	2.58713	3.38617
Best	2.55281	4.39728	2.83857	2.88402	2.49226	2.91938
Worst	2.81524	4.90177	3.52762	3.20579	2.85774	3.85635
Std.	0.00428103	0.010446	0.0204176	0.00886905	0.00533416	0.0688828

**Table 18:** The result of environment 10.

	FWOA	PSO	WOA	HS	FA	MSA
Mean	2.62072	3.3095	3.75894	3.31711	3.38436	3.58167
Best	2.59849	3.09663	3.34706	3.00046	3.09922	3.37216
Worst	2.72948	3.59165	4.23291	3.59857	3.66757	4.15024
Std.	0.00127214	0.0167968	0.0506245	0.0199517	0.0203131	0.0265063

**Table 19:** The *p*-value.

	Environment 6	Environment 7	Environment 8	Environment 9	Environment 10
PSO vs. FWOA	4.81232 × 10^−310^	0	0	1.05172 × 10^−252^	2.49163 × 10^−301^
WOA vs. FWOA	9.88131 × 10^−324^	0	0	5.57775 × 10^−315^	0
HS vs. FWOA	1.60769 × 10^−320^	0	0	7.70742 × 10^−322^	1.84425 × 10^−318^
FA vs. FWOA	3.15265 × 10^−318^	0	0	2.30662 × 10^−276^	3.30036 × 10^−321^
MSA vs. FWOA	7.88286 × 10^−308^	1.15611 × 10^−321^	0	3.95253 × 10^−323^	1.22528 × 10^−321^

## Data Availability

The data presented in this study are available on request from the corresponding author.
